# Neuronal hypofunction and network dysfunction in a mouse model at an early stage of tauopathy

**DOI:** 10.1002/alz.14273

**Published:** 2024-10-05

**Authors:** Changyi Ji, Xiaofeng Yang, Mohamed Eleish, Yixiang Jiang, Amber M. Tetlow, Soomin C. Song, Alejandro Martín‐Ávila, Qian Wu, Yanmei Zhou, Wenbiao Gan, Yan Lin, Einar M. Sigurdsson

**Affiliations:** ^1^ Department of Neuroscience and Physiology Neuroscience Institute, New York University Grossman School of Medicine New York USA; ^2^ Department of Pathology New York University Grossman School of Medicine New York USA; ^3^ IonLab New York University Grossman School of Medicine New York USA; ^4^ Skirball Institute New York University Grossman School of Medicine New York USA; ^5^ Department of Psychiatry New York University Grossman School of Medicine New York USA

**Keywords:** calcium imaging, immunotherapy, mouse model, network dysfunction, neuronal hypofunction, tau protein, tauopathy, two‐photon imaging

## Abstract

**INTRODUCTION:**

It is unclear how early neuronal deficits occur in tauopathies, if these are associated with changes in neuronal network activity, and if they can be alleviated with therapies.

**METHODS:**

To address this, we performed in vivo two‐photon Ca^2+^ imaging in tauopathy mice at 6 versus 12 months, compared to controls, and treated the younger animals with a tau antibody.

**RESULTS:**

Neuronal function was impaired at 6 months but did not deteriorate further at 12 months, presumably because cortical tau burden was comparable at these ages. At 6 months, neurons were mostly hypoactive, with enhanced neuronal synchrony, and had dysregulated responses to stimulus. Ex vivo, electrophysiology revealed altered synaptic transmission and enhanced excitability of motor cortical neurons, which likely explains the altered network activity. Acute tau antibody treatment reduced pathological tau and gliosis and partially restored neuronal function.

**DISCUSSION:**

Tauopathies are associated with early neuronal deficits that can be attenuated with tau antibody therapy.

**Highlights:**

- Neuronal hypofunction in awake and behaving mice in early stages of tauopathy.
- Altered network activity disrupted local circuitry engagement in tauopathy mice.
- Enhanced neuronal excitability and altered synaptic transmission in tauopathy mice.
- Tau antibody acutely reduced soluble phospho‐tau and improved neuronal function.

## 1 BACKGROUND

Accumulation of hyperphosphorylated tau is a major pathological hallmark of Alzheimer's disease and frontotemporal dementia with tauopathy.^1^ In healthy neurons, tau plays important roles in supporting a variety of neuronal functions, such as axonal transport and synaptic signaling.^2^ Under pathological conditions, excessive post‐translational modifications of tau lead to its misfolding and aggregation, and thereby compromise normal neuronal function.^3^

Previous studies have reported abnormal neuronal function in tauopathy mouse models using in vivo two‐photon Ca^2+^ imaging.^4^, ^5^, ^6^ Two of them reported a decrease in neuronal activity, as measured by Ca^2+^ transient frequency and total Ca^2+^ activity in two different tauopathy mouse models under anesthesia or quiet wakefulness.^5^, ^6^ Our previous study also detected altered neuronal activity in behaving JNPL3 tauopathy mice during resting or treadmill running.^4^ The enhanced neuronal activity during running led to more pronounced abnormal Ca^2+^ profiles compared to resting mice. The mice used in that study were 10‐ to 12‐months‐old and had moderate behavior and motor deficits. In JNPL3 mice, tau pathology starts to accumulate at 4–6 months and progresses relatively slowly compared to some other tauopathy mouse models.^7^ Thereby, it allows a wider time window to study tau pathogenesis and the progression of its pathology, and to examine the therapeutic efficacy of potential disease‐modifying drugs. Because the presence of pathological tau may compromise neuronal function much earlier than the onset of behavior deficits, we sought to identify early neuronal deficits in young JNPL3 mice at the onset of tau pathology at 6 months of age, compared to mice at 12 months of age.

**RESEARCH IN CONTEXT**

1. **Systematic review**: The authors reviewed the literature using PubMed and Google Scholar. There are limited prior in vivo two‐photon Ca^2+^ imaging studies in awake and behaving tauopathy mice. The relevant citations are cited appropriately. In addition, it is unclear how early neuronal deficits occur in tauopathies, and if they can be alleviated with therapies. Finally, neuronal network activity has not been well studied in behaving tauopathy models.
2. **Interpretation**: Our findings reveal early functional neuronal and network deficits in tauopathies that can be partially attenuated with acute tau antibody therapy.
3. **Future directions**: The manuscript highlights the importance of analyzing neuronal function and network activity at different ages in tauopathies, and supports chronic studies on this particular antibody that is likely to lead to improved efficacy. How neuronal function relates to soluble versus insoluble tau and treatment effects on these different parameters provides valuable insight into tau pathogenesis and should ideally be a common readout in such studies.

Different types of neurons exhibit distinct patterns of activity during motor behaviors. For example, some neurons are activated, whereas others are suppressed during running.^8^, ^9^ This segregation of neuronal firing patterns underlies circuitry mechanisms that are critical for information processing, movement initiation, and execution.^8^, ^9^, ^10^ Neuronal network function can involve rhythmic and synchronized firing,^11^ and coherent patterns are essential for cognitive function.^11^ However, excessive synchronization may be pathological, as seen in seizures associated with epilepsy and Alzheimer's disease.^12^, ^13^ It remains unclear whether and how tau pathology affects neuronal network activity. During motor behaviors, excitatory pyramidal neurons in the primary motor cortex increase their activity and exhibit sequential activation, that is stabilized after motor learning.^8^, ^9^, ^10^ Inhibitory neurons display more diverse responses,^8^, ^14^ and inhibition shapes the activity profiles of pyramidal neurons by tuning their responsiveness to motor stimuli.^8^, ^10^, ^14^ Whether the neuronal firing pattern during motor behaviors changes under pathological conditions like tauopathy warrants further investigation.

**FIGURE 1:**
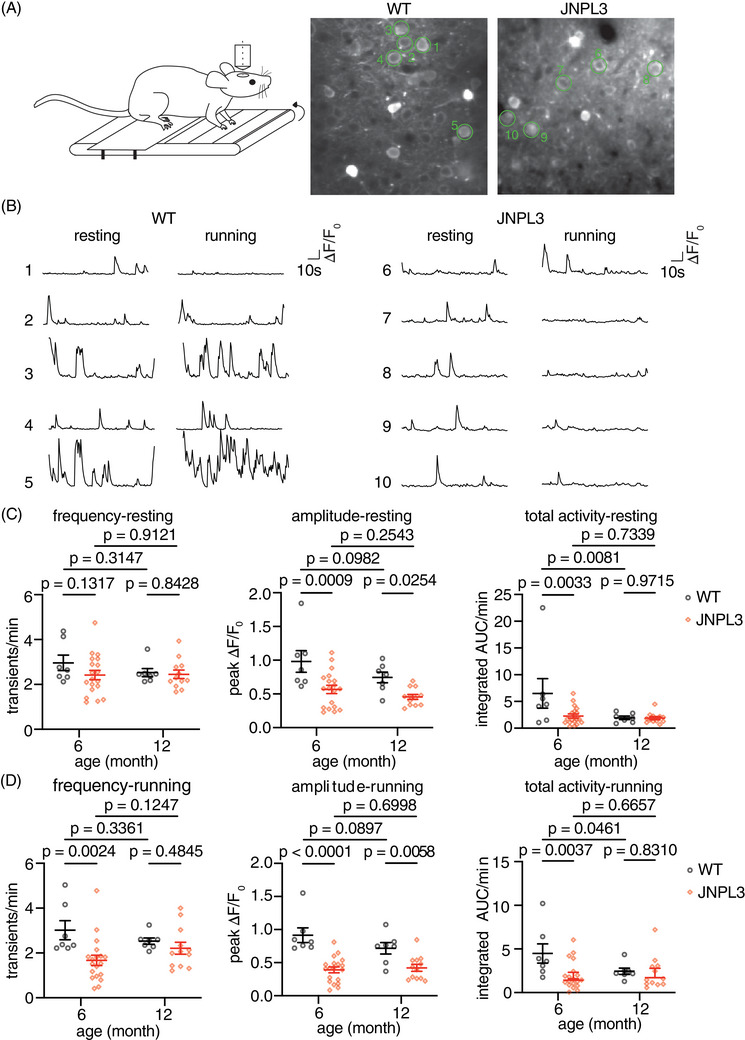
Decreased somatic Ca^2+^ activity in L2/3 motor cortex in 6‐month‐old JNPL3 mice at early stage of tauopathy. (A) Schematic representation of two‐photon imaging in head‐fixed, awake, and behaving mice. GCaMP6s was expressed in motor cortex layer 2/3 neurons. Representative images of AAV‐syn‐GCaMP6s expression are shown in the right panel with green circles outlining analyzed neurons. (B) Representative Ca^2+^ transient traces in motor cortex layer 2/3 neurons when mice were either resting or running. F_0_: fluorescence intensity at baseline, ΔF: fluorescence intensity per time point minus fluorescence intensity at baseline. (C) Ca^2+^ activity frequency (transients/min), Ca^2+^ amplitude (peak ΔF/F_0_), and total Ca^2+^ activity (integrated AUC/minute) were analyzed in 6‐month or 12‐month‐old mice when animals were resting on the treadmill. (D) The same parameters were analyzed in the same 6‐ and 12‐month‐old mice when animals were forced to run on the treadmill. The 6‐month‐old group consisted of 7 WT and 19 JNPL3 mice, and the 12‐month‐old group consisted of 7 WT and 12 JNPL3 mice. Each data point represents one animal. Two‐way analysis of variance (ANOVA) with Fisher's LSD multiple comparisons test in (C) and (D). AAV, adeno‐associated virus; AUC, area under the curve; WT, wild‐type.

In this study, we performed in vivo two‐photon Ca^2+^ imaging in 6‐ and 12‐month‐old JNPL3 mice and their age‐matched wild‐type (WT) controls when mice were either awake and resting, or performing a running task on the treadmill (Figure 1A,B). In 6‐month‐old mice, we observed a significant decrease of neuronal activity in JNPL3 mice compared to WT controls, as indicated by the frequency of Ca^2+^ transients in the running condition, as well as by Ca^2+^ transient amplitude and integrated total Ca^2+^ activity in both resting and running conditions. In 12‐month‐old cohorts, only Ca^2+^ transient amplitude decreased significantly in JNPL3 mice compared to age‐matched WT controls in both resting and running conditions. In addition, an age‐dependent decrease of total Ca^2+^ activity was observed in WT mice, but not in JNPL3 mice. Furthermore, we examined Ca^2+^ activity profiles and neuronal population activity in the younger cohort, which was not explored in our previous study.^4^ The proportion of hypoactive neurons was significantly larger in JNPL3 mice, and running‐related responses were largely suppressed. This reduction in neuronal Ca^2+^ activity was likely associated with decreased excitatory synaptic connections. Nevertheless, motor cortical neurons in JNPL3 mice exhibited higher synchronization compared to that in WT mice. This change might be related to enhanced inhibitory neuronal activity. Finally, acute treatment of the tauopathy mice with a tau monoclonal antibody partially restored the decreased neuronal activity but did not alter network activity, and the antibody decreased soluble phosphorylated tau (p‐tau), increased insoluble total tau, and attenuated gliosis in the mice.

## 2 METHODS

### 2.1 Animals

Homozygous transgenic JNPL3 mice expressing human 0N4R tau with P301L mutation and age‐matched wild‐type (WT) mice of the same strain background at 6 and 12 months of age were used in this study.^7^ Human tau is expressed at approximately twice the levels of mouse tau in homozygous JNPL3 mice.^7^ We chose female mice as in our previous study because they have more tau pathology than male mice.^4^ All animals were housed at New York University (NYU) Grossman School of Medicine animal facilities. All procedures were approved by the Institutional Animal Care and Use Committee (IACUC) of the university, and are in accordance with National Institutes of Health (NIH) Guidelines, which meet or exceed the ARRIVE guidelines (Animal Research: Reporting of In Vivo Experiments).

### 2.2 Surgery

Cranial window surgery was performed as we have described previously.^4^ Briefly, the mouse was placed in a stereotaxic frame with a heated pad under isoflurane anesthesia. A round opening of 3 mm in diameter was drilled into the skull over the right primary motor cortex. Subsequently, a total of 0.6 µL of adeno‐associated virus (AAV) 5‐Syn‐GCaMP6s virus (1.8 × 10^13^ genome copies per mL, Addgene) was slowly injected by Nanoject III Nanoliter Injector into layer 2/3 motor cortex (1.5 mm anterior from bregma, 1.5 mm lateral from midline) to fluorescently label the neurons. Then the skull opening was covered by a round coverslip. Dental cement was used to seal the edges of the cover glass and embed a head holder composed of two parallel micro‐metal bars. Mice were individually housed and allowed to recover for 4 weeks before two‐photon imaging.

### 2.3 In vivo two‐photon microscopy

A custom‐built free‐floating treadmill was placed under the microscope. Mice were head‐fixed and allowed to move their forelimbs on the treadmill to perform a running task. Mice were first placed on the treadmill to record the Ca^2+^ activity in a quiet restful state (referred to as resting) for 100 s. Then the treadmill was turned on and the mice were forced to run at a speed of 1.67 cm/s (referred to as running). When the motor was turned on, the belt speed of the treadmill increased gradually from 0 cm/s to 1.67 cm/s within 2 s. Each mouse ran five trials with a short resting period (25 s) in‐between trials. Each running trial lasted for 100 s.

Two‐photon imaging was collected with an Olympus Fluoview 1000 two‐photon system (920 nm) equipped with a Ti:Sapphire laser (MaiTai DeepSee, Spectra Physics). Ca^2+^ signals were recorded at 2 Hz using a 25× objective (NA 1.05), with a frame size of 320 × 256 pixels with a 1× digital zoom, or 256 × 256 pixels with a 2× digital zoom. Images were taken at a depth of 200–300 µm below the pial surface. The same focal plane with the same neurons was imaged again after antibody treatment. Images were acquired by Fluoview software and analyzed post hoc using ImageJ, MATLAB, Python, and R/RStudio.

### 2.4 Image analysis

Individual images were first motion‐corrected in Fiji ImageJ using the plugin Image Stabilizer (https://imagej.net/Fiji). Subsequently, all images from different trials of the same animals were aligned by the motion correction function of Using_the_Acquisition2P_Class.m in MATLAB (https://github.com/HarveyLab/Acquisition2P_class/tree/master). Once the trial alignments were finished, all the images from the same animals were concatenated chronologically into a single image in ImageJ. This image was then analyzed using the Mesmerize package.^15^ Regions of interest (ROIs) corresponding to neuronal somas and the time‐dependent component of relative fluorescence change df/f within each ROI were extracted using CaImAn toolbox in Mesmerize. The CNMF method in the CaImAn toolbox allows for elimination of signal contamination from the background and nearby neuropils.^16^ Subsequently, df/fs were exported from Mesmerize for further analyses.

The frequency of Ca^2+^ transients, amplitude of transients, and total Ca^2+^ activity were analyzed in a custom‐written R script. Briefly, a time window spanning 15% of the trace length was slid across the trace, and the trace with minimal standard deviation (SD) within a window was selected as baseline. The threshold was set as the average of baseline plus five times its SD. The frequency of Ca^2+^ transients was calculated by counting the number of Ca^2+^ transients per minute for each soma. The peak amplitude was defined as the highest value of the Ca^2+^ transients in each trace. The mean amplitude was the average of all Ca^2+^ transients in a given trace. Finally, the total Ca^2+^ activity was quantified by calculating the area under the curve of Ca^2+^ transients per minute.

To analyze running‐related responses, the Ca^2+^ activity (df/f) of each neuron was averaged across five trials. The averaged trace of each neuron was then aligned to the time when the treadmill started. Neurons were categorized into different groups by comparing Ca^2+^ activity (df/f) before and after the start of the treadmill (stationary vs moving). Specifically, the baseline activity of each neuron was calculated by averaging its activity during resting and was subtracted from the averaged df/f during running. After the treadmill started, if the baseline‐subtracted activity deviated more than three times the SD of baseline for at least three consecutive frames (equivalent to 1.5 s), the neuron was identified as one that responded to running. The neuron was further categorized as “activated” or “suppressed” if the df/f was above or below the baseline, respectively. When deviations occurred in both directions, the neuron was categorized as a mixed‐response neuron. The rest of the neurons showed no change between resting and running.

Pearson's correlation coefficient of Ca^2+^ activity (df/f) was calculated between each pair of neurons for each field of view. The mean correlation value of a given cell was averaged from all the correlation coefficients between that cell and all other simultaneously recorded cells within the same field of view. The correlation matrices were calculated separately when animals were either resting or running and averaged from five trials.

### 2.5 In vitro brain slice recordings

WT or JNPL3 female mice (6‐ to 7‐months‐old) were first anesthetized with isoflurane and then decapitated. Brains were quickly removed and immersed in ice‐cold oxygenated cutting solution containing (in mM): 87 NaCl, 2.5 KCl, 1.25 NaH_2_PO_4_, 25 NaHCO_3_, 10 glucose, 75 sucrose, 1.3 ascorbic acid, 7 MgCl_2_, and 0.5 CaCl_2_. Coronal slices (350 μm) were cut with a vibrating microtome (Leica) and immediately transferred to a chamber with oxygenated (95% O_2_/5% CO_2_) artificial cerebrospinal fluid (aCSF) containing (in mM): 124 NaCl, 2.5 KCl, 1.25 NaH_2_PO_4_, 26 NaHCO_3_, 10 glucose, 1.5 MgCl_2_, and 2.5 CaCl_2_ at 35°C for 20 min. The chamber with slices was then transferred to room temperature to allow the slices to recover for at least 1 h. For recording, slices were placed in a recording chamber perfused with oxygenated aCSF that was heated to 30°C. The recording chamber was held under an Olympus microscope equipped with infrared differential interference optics. Chemicals were all purchased from Sigma‐Aldrich unless stated otherwise.

Whole‐cell recording was performed on individual neurons within layers 2/3 of the primary motor cortex. Patch pipettes were pulled from borosilicate glass (World Precision Instruments, 140N‐15) with an access resistance of 3–5 MΩ. These pipettes were filled within an internal solution containing (in mM): 127 K‐gluconate, 8 KCl, 10 phosphocreatine, 10 HEPES, 4 Mg‐ATP, and 0.3 Na‐GTP (pH 7.3, 280 mOsm). After membrane breaking to form whole cell configuration, the resting membrane potential was measured for each neuron. Subsequently, action potentials (APs) were stimulated by injecting a series of depolarization current steps from a holding potential of ‐70 mV. Data were recorded at a sampling rate of 20 kHz and filtered at 2 Hz by an 8‐pole Bessel filter using a MultiClamp 200 amplifier (molecular devices). Cells with resting membrane potential more depolarized than ‐50 mV and access resistance exceeding 25 MΩ were excluded from further analyses. Fast‐spiking (FS) or non‐FS cells were identified based on their AP firing patterns. AP waveforms were analyzed by custom‐written Matlab scripts.

To record spontaneous excitatory postsynaptic current (sEPSC), the internal solution to record AP was used. The cell types were first identified by AP firing patterns and then followed by sEPSC recording. The membrane potential was held at ‐70 mV, which was at the reversal potential of Cl^−^. Data were analyzed in Clampfit (Molecular Devices).

### 2.6 Antibodies and intravenous injections

Mouse monoclonal tau antibody 8B2 IgG1κ was generated by GenScript as described previously by immunizing BALB/c mice with a peptide encompassing the phospho‐Ser396/404 (p‐tau‐Ser396/404) region of the tau protein that was conjugated to keyhole limpet hemocyanin via a cysteine residue (cTDHGAEIVYK(pS)PVVSGDT(pS)PRHL).^17^ Hybridoma fusions were screened by enzyme‐linked immunosorbent assay (ELISA) and 8B2 was selected with other antibodies based on their binding to the p‐tau peptide immunogen. We have reported previously on its crystal structure and binding characteristics.^17^ IgG1κ (referred to as IgG1, eBioscience, 16‐4714) was used as a control of the same subclass. IgG1 or 8B2 antibodies (100 µg per injection) were injected intravenously into JNPL3 mice on Day 1 and Day 4 after the initial image session (Day 0). The dose of each injection equals about 3 mg/kg antibody. A subset of mice was injected intravenously with antibodies labeled with VivoTag 680XL to assess target engagement.

### 2.7 Tissue processing

After imaging, the brains were collected for downstream analyses. Briefly, mice were perfused with phosphate‐buffered saline (PBS) before brain extraction. The right hemispheres were fixed in 4% paraformaldehyde at 4°C overnight and then transferred into 2% dimethylsulfoxide (DMSO) and 20% glycerol in phosphate buffer for long‐term storage at 4°C until cryosection. Brain slices were sectioned coronally at 40 µm for immunohistology. The left brain hemispheres were flash‐frozen on dry ice and stored at ‐80°C for subsequent protein assays and biochemical analyses.

### 2.8 Western blot

The left brain hemispheres were homogenized in ice‐cold modified radioimmunoprecipitation assay (RIPA) buffer (50 mM Tris‐HCl, 150 mM NaCl, 1 mM ethylenediaminetetraacetic acid (EDTA), 1% Nonidet P‐40, 0.25% sodium deoxycholate, pH 7.4) with protease cocktail (cOmplete, Roche) and phosphatase inhibitor cocktail (1 mM NaF, 1 mM Na_3_VO_4_, 1 nM phenylmethylsulfonyl fluoride (PMSF). The brain homogenates were centrifuged at 20,000 × *g* for 1 h at 4°C. The resulting supernatants were collected as low‐speed supernatant (LSS). To isolate the sarkosyl insoluble fraction, 2 mg total protein from LSS was diluted in RIPA buffer with 1% sarkosyl, and then centrifuged at 100,000 × *g* for 1.5 h at 20°C. The pellet was washed by RIPA buffer with 1% sarkosyl and centrifuged again at 100,000 × *g* for 1 h at 20°C. The sarkosyl pellet (SP) was air dried for 30 min, and then dissolved in 50 µL of 5× sample buffer (62.5 mM Tris‐HCl, 10% glycerol, 5% β‐mercaptoethanol, 2.3% sodium dodecyl sulfate (SDS), 1 mM EDTA, and 1 mM ethylene glycol‐bis(β‐aminoethyl ether) tetraacetic acid (EGTA). The LSS was eluted with 1× sample buffer. All samples were boiled for 10 min, followed by electrophoresis on 12% SDS‐polyacrylamide gel electrophoresis (PAGE) gel and transferred onto nitrocellulose membrane. The blots were blocked by 5% milk in Tris buffered saline with 1% TritonX‐100 (TBST). Primary antibodies, including PHF1 (1:1000, gift from Peter Davies), CP27 (1:500, gift from Peter Davies), pT231 (1:2000, Thermo Fisher Scientific, 1H6L6), AT8 (1:100, Thermo Fisher Scientific, MN1020), glyceraldehyde‐3‐phosphate dehydrogenase (GAPDH, 1:5000, Cell Signaling Technology, D16H11), glial fibrillary acidic protein (GFAP, 1:2000, Bioss, bs0119R), and ionized calcium‐binding adaptor molecule 1 (Iba1, 1:2000, Fujifilm Wako, 019‐19741) were diluted in Superblock (Thermo Fisher Scientific), and incubated with the membrane at 4°C overnight. The following day the membrane was incubated with diluted (1:10,000) IRDye 800CW goat anti‐rabbit or IRDye 680RD goat anti‐mouse secondary antibodies (LICOR Biosciences). Blots were then detected by Odyssey CLx imaging system (LICOR Biosciences).

### 2.9 Immunohistochemistry

Brain sections were washed in PBS, followed by permeabilization and blocking in PBS with 5% bovine serum albumin, 2% normal goat serum, and 0.3% Triton‐X‐100 at room temperature for 3 h. Subsequently, the sections were incubated with diluted primary antibodies in PBS with 5% bovine serum albumin and 1% normal goat serum at 4°C overnight. The dilution of the primary antibodies was as follows: PHF1 (1:100, gift from Peter Davies), CP27 (1:100, gift from Peter Davies), NeuN (1:200, Cell Signaling Technologies, D4G4O), GFAP (1:200, Bioss, bs0119R), and Iba1 (1:200, Fujifilm Wako, 019‐19741). On the following day, the brain sections were incubated with diluted secondary antibodies (1:1000) goat anti‐mouse Alexa Fluor 568 or goat anti‐rabbit Alexa Fluor 568 at room temperature for 3 h. After washing, the sections were mounted in ProLong Gold antifade reagent (Thermo Fisher Scientific, P10144), and imaged by an LSM 700 Zeiss confocal laser scanning microscope. Images were processed by Fiji ImageJ software.

### 2.10 Statistics

Statistics were performed using GraphPad Prism 9. Data are presented as mean ± standard error of the mean (SEM). Before applying statistical comparisons, data distributions were assessed by the Shapiro–Wilk normality test. For normally distributed data, paired or unpaired *t*‐tests were used, whereas non‐normally distributed data were analyzed using the Mann–Whitney *U* test or Kolmogorov–Smirnov test. The specific tests used for each figure are indicated in the corresponding figure legend and summarized in Table S1. All comparisons were two‐tailed, and a significance level was set to *p* ≤ 0.05.

## 3 RESULTS

### 3.1 Altered somatic Ca^2+^ activity in JNPL3 mice during the early versus later stage of tauopathy

First we determined if Ca^2+^ abnormalities can be detected before the development of robust tau pathology with neurofibrillary tangles.^7^ We followed the similar experimental paradigm as described in our previous study^4^ and performed in vivo two‐photon Ca^2+^ imaging in L2/3 motor cortical region when animals were resting or forced to run on a treadmill. We analyzed neuronal Ca^2+^ profiles in both 6‐ and 12‐month‐old JNPL3 mice, and compared then to their age‐matched wild‐type (WT) controls.

In resting 6‐month‐old mice, the amplitude of Ca^2+^ transients and total Ca^2+^ activity decreased significantly in JNPL3 mice compared to age‐matched WT controls. However, the frequency of somatic Ca^2+^ transients did not change (Figure 1C). When these mice were running on the treadmill, all these three parameters decreased significantly in JNPL3 mice compared to WT controls (Figure 1D). In 12‐month‐old JNPL3 mice, only the amplitude of Ca^2+^ transients decreased under both resting and running conditions, whereas the frequency of Ca^2+^ transients and total Ca^2+^ activity did not differ significantly between JNPL3 and age‐matched WT mice (Figure 1C,D). Of interest, the total Ca^2+^ activity decreased significantly in 12‐month‐old WT mice compared to 6‐month‐old WT mice. However, no significant difference was observed in other Ca^2+^ activity parameters between these two age groups within either the WT or JNPL3 groups. Overall, awake and behaving JNPL3 mice at an early stage of tauopathy exhibited a pronounced decrease of neuronal activity in the motor cortex compared to age‐matched WT mice. This attenuation of neuronal activity did not deteriorate further from 6 to 12 months of age.

JNPL3 mice were reported originally to develop tau tangle pathology at about 10 months, mainly in subcortical regions, in particular brainstem and spinal cord. At this age, pre‐tangle pathology is observed in cortex, hippocampus, and basal ganglia. Because the neuronal Ca^2+^ activity is being analyzed in the motor cortex, we focused on examining tau pathology within that region by immunohistochemistry and in forebrain cortex by western blots. In the low speed supernatant (LSS) of forebrain cortical homogenates, we confirmed the expression of human tau (recognized by CP27 antibody) and the presence of phosphorylated tau (p‐tau) Thr231 (pT231), p‐tau Ser202/Thr205 (pS202/T205, AT8), and p‐tau Ser396/Ser404 (pS396/S404, PHF1) in both 6‐ and 12‐month‐old JNPL3 mice (Figure S1A,B, complete blots in Figure S2). The total tau and pT231 LSS levels were similar in 6‐ and 12‐month‐old JNPL3 mice. However, pS202/T205 levels increased, whereas pS396/S404 levels decreased in the 12‐month‐old compared to the 6‐month‐old cohort. Of interest, levels of sarkosyl insoluble human tau in the forebrain cortex did not change from 6 to 12 months of age (Figure S1C,D, complete blot in Figure S2). We further confirmed the presence of pre‐tangle pathology in the primary motor cortex layer 2/3 region of JNPL3 mice by immunostaining (Figure S1E–H). In 6‐month‐old JNPL3 mice, AT8, MC1, and PHF1 staining was sparse but increased at 12 months of age. Taken together, changes in LSS tau in forebrain cortex varied in 6‐ versus 12‐month‐old mice depending on the epitope, whereas levels of sarkosyl insoluble tau were comparable at these ages. In addition, p‐tau immunoreactivity in the motor cortex increased in older animals. The lack of increase in overall cortical tau burden from 6 to 12 months may explain why neuronal activity did not deteriorate from 6 to 12 months.

### 3.2 Neuronal hypofunction in 6‐month‐old JNPL3 mice

**FIGURE 2:**
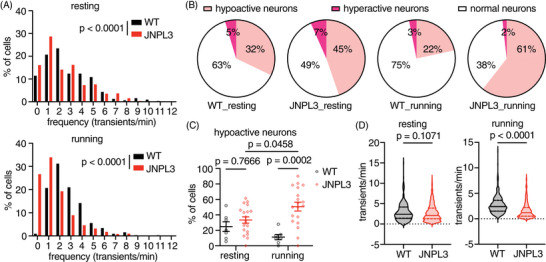
Increased hypoactive neurons in L2/3 motor cortex in running JNPL3. (A) Further analysis of all neurons showed altered frequency distribution of Ca^2+^ transients in JNPL3 mice when animals were either resting or running on the treadmill, compared to WT mice. (B) The fraction of hypoactive neurons increased in both resting and running JNPL3 mice, compared to WT mice. Hypoactive neurons: <2 transients/min; hyperactive neurons: >6 transients/min; and normal neurons: 2–6 transients/min. A total of 325 neurons from 7 WT animals and 852 neurons from 19 JNPL3 mice were analyzed. (C) The fraction of hypoactive neurons in each animal (represented by a data point). (D) Ca^2+^ transient frequency in active neurons (>0 transients/min). While resting, a total of 311 neurons from WT animals and 737 neurons from JNPL3 mice were analyzed. While running, a total of 325 neurons from WT animals and 800 neurons from JNPL3 mice were analyzed. Two‐way ANOVA with Tukey's multiple comparisons test in (C). Mann–Whitney *U* test in (D). WT, wild‐type.

To gain further insight into abnormal neuronal activity observed in 6‐month‐old JNPL3 mice, we looked into the pattern of Ca^2+^ activity at the neuronal level, which had not been examined thoroughly in our previous study.^4^ There was a significant change in the distribution of Ca^2+^ transient frequency in JNPL3 mice compared to WT mice, during both resting and running conditions (Figure 2A). We classified these neurons into three categories based on their Ca^2+^ activity frequency. Neurons exhibiting fewer than two transients per minute were considered to be hypoactive, whereas those with more than six transients per minute were categorized as hyperactive.^6^, ^18^, ^19^, ^20^ Neurons with two to six transients per minute were considered to have normal activity.

In resting WT mice, 32% of neurons were hypoactive, 63% were normal, and 5% were hyperactive (Figure 2B). When these mice were running, 22% of neurons were hypoactive, 75% had normal activity, and 3% were hyperactive (Figure 2B). In age‐matched JNPL3 mice, the fraction of hypoactive neurons increased to 45%, and hyperactive neurons increased to 7% when animals were at rest (Figure 2B). As a result, the fraction of neurons with normal activity decreased to 49% (Figure 2B). When JNPL3 mice were running on the treadmill, the fraction of hypoactive neurons increased further to 61%, hyperactive neurons decreased to 2%, and normal neurons went down to 38% (Figure 2B).

We further analyzed the fraction of hypoactive neurons in each animal to confirm that this result was not confounded by dominant changes in certain animals. Consistently, running JNPL3 mice had a larger fraction of hypoactive neurons than running WT controls (JNPL3 vs WT: 51% vs 11%). Moreover, the fraction of hypoactive neurons in JNPL3 mice during running was also larger than the fraction when these animals were at resting state (running vs resting: 51% vs 33%) (Figure 2C). The average frequency of Ca^2+^ transients in active neurons decreased in running but not in resting JNPL3 mice, compared to WT controls (Figure 2D).

Consistent with our previous study in 12‐month‐old animals,^4^ the peak amplitude of Ca^2+^ transients and total Ca^2+^ activity in active neurons decreased in 6‐month‐old JNPL3 mice compared to WT controls, when animals were either resting or running on the treadmill (Figure S3A,C,E,G). There was a left shift of cumulative frequency distribution of peak Ca^2+^ amplitude and total Ca^2+^ activity (Figure S3B,D,F,H). Taken together, JNPL3 mice had lower neuronal activity compared to their age‐matched WT controls (see Table S2 for summary).

### 3.3 Altered neuronal network activity in 6‐month‐old JNPL3 mice

**FIGURE 3:**
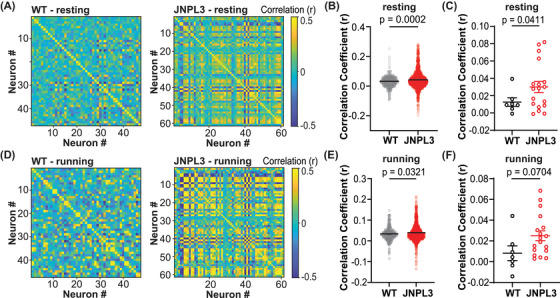
Increased neuronal activity synchrony in L2/3 motor cortex in JNPL3 mice. (A) Correlation coefficient matrix of Ca^2+^ activity in a resting WT and JNPL3 mouse. In a given FOV, Ca^2+^ activity from every two neurons was compared in pairs, and assessed by Pearson's correlation coefficient. (B) Ca^2+^ activity correlation in all neurons in resting animals. A correlation index was calculated for each neuron by averaging the Pearson's correlation coefficient of a given neuron with all other neurons in the same FOV. Each dot represents a neuron. A significant increase in Ca^2+^ activity correlation was observed in resting JNPL3 mice compared to WT mice. (C) Ca^2+^ activity correlation in resting WT and JNPL3 mice. Each dot represents the mean correlation index of all neurons in a given animal. (D) Same analysis as (A) in a running WT and JNPL3 animal. (E) Same analysis as (B) in running animals. Ca^2+^ activity correlation significantly increased in running JNPL3 mice compared to WT controls. (F) Same analysis as (C) in running animals. Pearson's correlations in (A) and (D). Unpaired *t*‐test in (B), (C), (E), and (F). FOV, field of view; WT, wild‐type.

Given the decreased neuronal activity in JNPL3 mice, we further assessed the impact of tau pathology on neuronal circuitry in primary motor cortex. We analyzed the correlations of neuronal Ca^2+^ dynamics, which reflect the strength of neuronal interactions within a given population.^5^, ^21^ First, we calculated Pearson's correlation coefficients between a given neuron and all other neurons in the field of view, which resulted in a correlation matrix (Figure 3A,D). Compared to WT mice, Ca^2+^ activity correlations were more prominent in JNPL3 mice during both resting and running periods (Figure 3A,D). Subsequently, we calculated a correlation index for a given neuron by averaging the Pearson's correlation coefficients of that neuron paired with all other neurons in the field of view. The correlation index increased significantly at the neuronal (Figure 3B) and animal (Figure 3C) levels in resting JNPL3 mice. A significant increase in the correlation index was also observed in running JNPL3 mice at the neuronal level (Figure 3E), but not at the animal level (Figure 3F). These results suggested increased synchrony of neuronal activity in JNPL3 mice, especially during the resting period.

**FIGURE 4:**
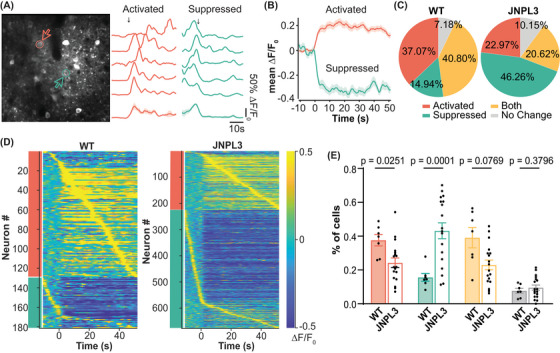
Altered neuronal responses to running in L2/3 motor cortical neurons in JNPL3 mice. (A) Representative Ca^2+^ transient traces from two neurons of a WT mouse showed either activated (red) or suppressed (green) activity in response to running. Ca^2+^ traces (normalized ΔF/F) from five trials were aligned to the onset of treadmill rotation as indicated by the arrow. The average trace of all five trials is shown at the bottom. The shade represents SEM. The vertical scale bar equals 50% maximum normalized ΔF/F_0_; horizontal scale bar represents 10 s. (B) Average traces of all neurons exhibiting activated or suppressed responses to running in WT mice. The solid line represents the mean value, and the shaded region shows SEM. (C) Neurons were categorized into four types according to their response to running. In WT mice (348 neurons, 7 animals), 37.1% of neurons were activated (red), 14.9% were suppressed (green), 40.8% showed both activated and suppressed responses (yellow), and 7.2% had no change (gray). In JNPL3 mice (975 neurons, 18 animals), 23.0% of neurons were activated, 46.3% were suppressed, 20.6% showed both activated and suppressed responses, and 10.2% showed no change. (D) Activity pattern of neurons with activated (red bar) or suppressed (green bar) response to running in WT (left) and JNPL3 (right) mice. The average activity of each neuron from five trials was aligned to the onset of the treadmill and then sorted by the time taken to their peak activity. (E) The fraction of each neuronal response type in each animal when mice were running. The ratio of activated neurons decreased while those with suppressed activity increased in JNPL3 mice compared to WT mice. Each dot represents an animal. Unpaired *t*‐tests. SEM, standard error of the mean; WT, wild‐type.

Different neurons respond to the same stimuli in different yet organized ways, which may reflect circuit mechanisms during information processing.^8^, ^10^ We next analyzed the responses of neuronal Ca^2+^ activity during running relative to resting (Figure 4A,B). Neurons were categorized into four types based on their responses to running relative to resting: (1) activated activity, (2) suppressed activity, (3) mixed response (both activated and suppressed activity), or (4) no change (Figure 4C). In WT mice, 37% of neurons were activated, 15% were suppressed, 41% had mixed responses, and 7% did not respond to running. In JNPL3 mice, 23% of neurons were activated, 46% showed suppressed activity, 21% had mixed responses, and 10% did not respond to running. In running JNPL3 mice compared to WT mice, the fractions of activated neurons decreased significantly, whereas the fractions of suppressed neurons increased significantly (Figure 4D,E). Although not statistically significant, there was a trend toward a decrease in the fraction of mixed‐responsive neurons in JNPL3 mice compared to WT mice (Figure 4E). In addition, the fraction of neurons that lacked a response to running remained the same between JNPL3 and WT mice (Figure 4E). Overall, neuronal activity was suppressed in running JNPL3 animals, which further supports the hypofunction of motor cortical neurons in JNPL3 tauopathy mice at 6 months of age.

**FIGURE 5:**
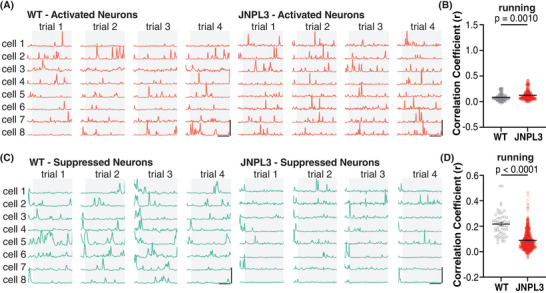
Differential changes of neuronal activity correlation in neurons with activated versus suppressed response to running in JNPL3 mice. (A) Representative Ca^2+^ trace of neurons with an activated response in a WT and a JNPL3 mouse in running trials. Gray background/box depicts running periods, whereas clear background/box denotes resting periods. (B) Ca^2+^ activity correlation in neurons with an activated response to running. The same analyses of correlation indexes were performed as in Figure 3B. A total of 129 neurons (7 animals) from WT and 224 neurons from JNPL3 (18 animals) were included. (C) Representative Ca^2+^ trace of neurons with a suppressed response during running in a WT and a JNPL3 mouse. (D) Ca^2+^ activity correlation in neurons with a suppressed response to running. The same analyses of correlation indexes were performed as in Figure 3B. A total of 52 neurons (7 animals) from WT and 451 neurons from JNPL3 (18 animals) were included. Unpaired *t*‐test in (H) and (J). WT, wild‐type.

Given the diverse responses of neuronal activity to running, Ca^2+^ activity correlation was assessed separately in neurons with either activated or suppressed activity when animals were running. In neurons that were activated during running, the Ca^2+^ activity correlation increased significantly in JNPL3 mice compared to WT mice (Figure 5A,B). In contrast, the correlation coefficient decreased significantly in neurons with suppressed Ca^2+^ activity when JNPL3 mice performed running tasks compared to WT mice (Figure 5C,D). Taken together, these data suggested that pathological tau altered the engagement pattern of local neuronal circuitry in the primary motor cortical L2/3 region of JNPL3 mice during running behaviors (see Table S2 for summary).

### 3.4 Increased neuronal excitability and changed synaptic transmissions in 6‐month‐old JNPL3 mice

**FIGURE 6:**
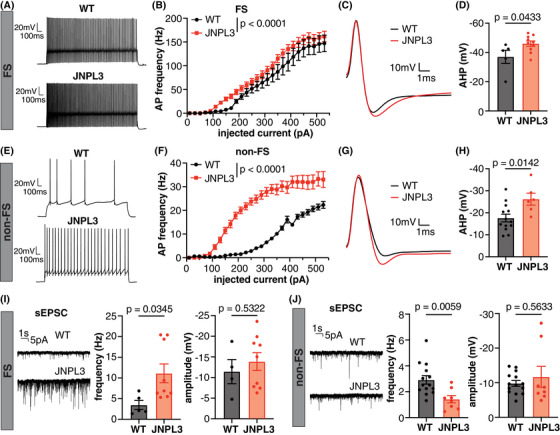
Increased excitability and altered excitatory synaptic transmission in L2/3 motor cortical neurons in JNPL3 mice. (A) Representative AP firing pattern in an FS neuron from a WT or JNPL3 mouse. Neurons were injected with a 290 pA depolarization current for 1 s. (B) F/I curve of FS neurons in WT or JNPL3 brain slices. Slight but significant left shift of F/I curve in JNPL3 FS neurons compared to WT controls. (C) Representative AP waveforms fired by FS neurons with minimal current injection. (D) AHP potential significantly increased in JNPL3 FS neurons, compared to WT controls. (E) Exemplar traces of AP firing pattern in non‐FS neurons injected with a 290 pA depolarization current for 1 s. (F) F/I curve of non‐FS neurons in WT or JNPL3 brain slices. Significant left‐shift of F/I curve in JNPL3 non‐FS neurons, compared to WT controls. (G) Representative AP waveforms fired by non‐FS neurons with minimal current injection. (H) AHP potential significantly increased in JNPL3 non‐FS neurons. (I) Frequency and amplitude of sEPSC in FS neurons. A significant increase of sEPSC frequency but not amplitude was observed in JNPL3 FS neurons. (J) Frequency and amplitude of sEPSC in non‐FS neurons. A significant decrease of sEPSC frequency but not amplitude was observed in JNPL3 non‐FS neurons. Two‐way ANOVA followed by Sidak's multiple comparisons test in (B) and (F). Unpaired *t‐*tests in (D), (H), (I), and (J). AHP, After‐hyperpolarization; AP, action potential; F/I, frequency current; FS, fast‐spiking; non‐FS, non‐fast‐spiking; sEPSC, spontaneous excitatory post‐synaptic current; WT, wild‐type.

To further dissect the mechanisms underlying the hypofunction of motor cortical neurons and disrupted network activity in JNPL3 mice, we assessed the intrinsic firing ability and connectivity of L2/3 motor cortical neurons through ex vivo patch‐clamp experiments. Neurons exhibiting fast‐spiking (FS) characteristics, which most likely were parvalbumin‐expressing interneurons, showed enhanced excitability in JNPL3 brain slices (Figure 6A,B). These neurons fired action potentials (APs) with larger afterhyperpolarization potential (AHP) compared to WT controls (Figure 6C,D). Other parameters related to intrinsic firing properties, such as input resistance, cellular capacitance, and rheobase current, were comparable between JNPL3 and WT mice (Table S3). Of interest, neurons without non‐FS properties demonstrated significantly increased excitability in JNPL3 mice compared to those in WT mice (Figure 6E,F). Similar to FS neurons, the amplitude of AHP in non‐FS neurons also increased in JNPL3 mice (Figure 6G,H). The resting membrane potential was depolarized in the non‐FS neurons of JNPL3 mice (WT, ‐75.07 ± 1.37 mV; JNPL3 mice, ‐69.07 ± 2.19 mV), which fired APs at lower rheobase currents (WT = 283.85 ± 24.74 pA; JNPL3 mice = 113.33 ± 18.91 pA). Consistently, non‐FS neurons in JNPL3 mice had decreased cellular capacitance and increased input resistance (Table S3). These non‐FS neurons were very likely pyramidal excitatory neurons given their abundance in the L2/3 motor cortex, although a minor fraction of them may be somatostatin or vasoactive intestinal peptide‐expressing inhibitory neurons. Overall, L2/3 motor cortical neurons exhibited enhanced intrinsic firing ability in JNPL3 mice compared to WT mice.

Furthermore, we assessed synaptic transmission in FS and non‐FS neurons by recording postsynaptic currents. The frequency of spontaneous excitatory post‐synaptic currents (sEPSCs) in FS neurons increased significantly in JNPL3 compared to WT mice (Figure 6I), suggesting enhanced excitatory synaptic transmission in these FS neurons in the presence of mutant tau. This may appear to contradict the two‐photon in vivo calcium findings that show hypofunction of layer 2/3 excitatory neurons. However, the layer 2/3 FS interneurons also receive excitatory inputs from other cortical layers^22^ and thalamocortical neurons.^23^ We speculate that these excitatory inputs from alternative sources contribute to the increased frequency of EPSCs in layer 2/3 FS interneurons. In contrast, the frequency of sEPSC in non‐FS neurons decreased significantly in JNPL3 mice compared to WT mice (Figure 6J), suggesting reduced excitatory transmission in these cells. The amplitude of sEPSC in either FS or non‐FS neurons remained similar between JNPL3 and WT mice.

Taken together, these data indicated impaired excitatory synaptic connections in non‐FS neurons in the L2/3 motor cortex of JNPL3 mice compared to WT mice, which may lead to homeostatic upregulation of their excitability.^24^ In contrast, FS neurons received enhanced excitatory synaptic transmission, which likely originated from brain regions that were outside the viewable area in the two‐photon in vivo studies, and that led to their own enhanced firing ability.

### 3.5 Reduced pathological tau and gliosis in 6‐month‐old JNPL3 mice after acute tau antibody 8B2 treatment

**FIGURE 7:**
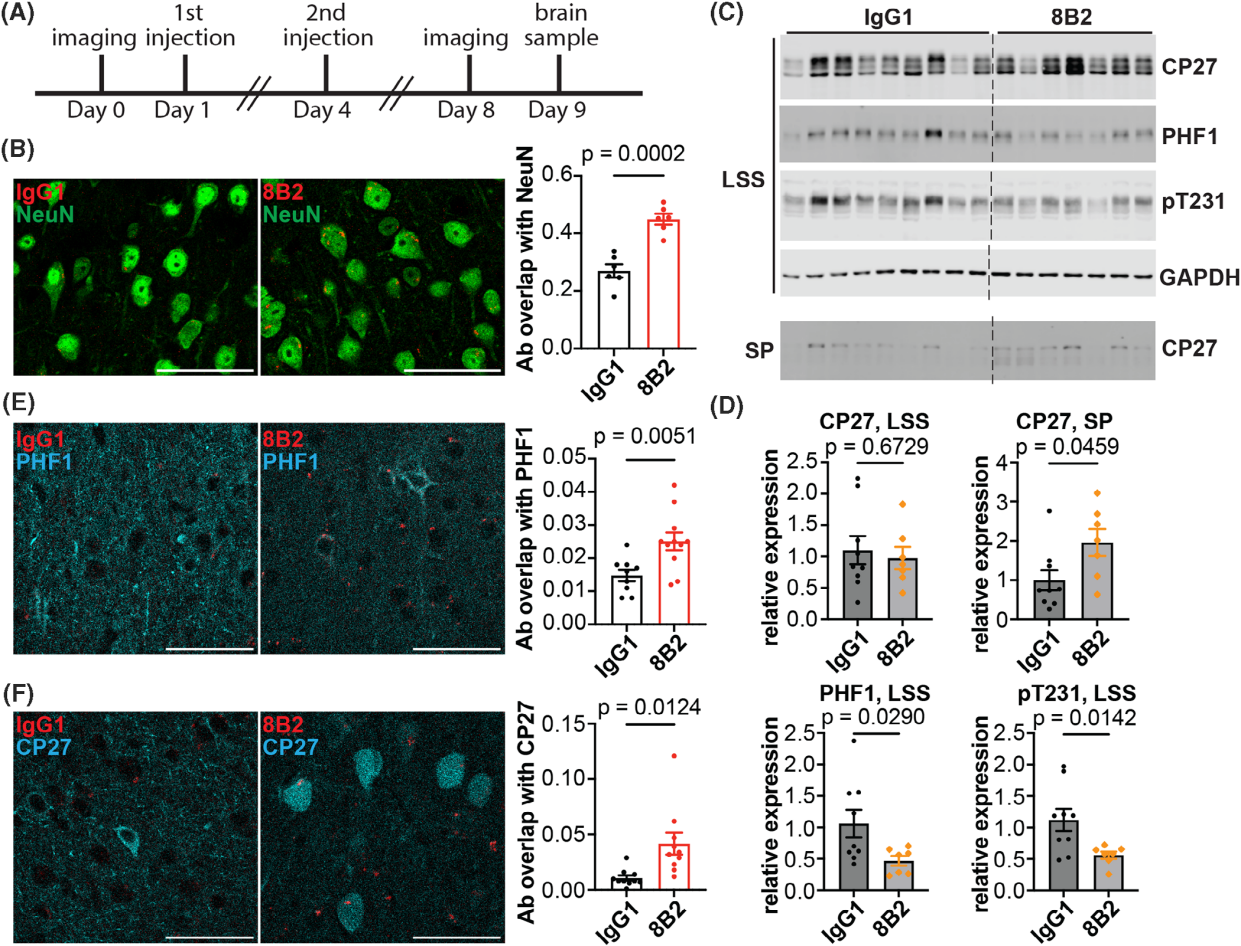
Tau antibody 8B2 is taken up into neurons, where it co‐localizes with tau and phosphorylated tau in the motor cortex of 6‐month‐old JNPL3 mice. (A) Schematic illustration of the imaging and antibody injection protocol. (B) Tau antibody 8B2 or control IgG1 labeled with VivoTag 680XL (red) were injected intravenously and the brains were extracted for analyses. Brain sections were stained with NeuN antibody. NeuN and 8B2 co‐localization was more prominent than NeuN and IgG1 colocalization. (C) Brain sections stained with PHF1 antibody. (D) Brain sections stained with CP27 antibody. More 8B2 was co‐localized with PHF1 or CP27 compared to control IgG1. (E) LSS protein fractions from brain lysates of the same JNPL3 mice as in (B) were blotted with CP27, PHF1, pT231, and GAPDH antibodies. The SP fraction of LSS was blotted with CP27. CP27 recognizes total human tau. PHF1 and pT231 recognize phosphorylated tau S396/S404 and T231, respectively. GAPDH is a loading control. (F) Tau protein quantification. LSS tau and p‐tau were normalized to GAPDH. SP tau was compared directly between the IgG1 and 8B2 treated groups. Unpaired *t*‐test in (B), (D), (E) and (F). Co‐localization was quantified by Mander's coefficient. Scale bar, 50 µm. LSS, low‐speed soluble; SP, sarkosyl pellet, insoluble protein.

A previous study from our group showed that a tau monoclonal antibody 4E6 that targets p‐tau‐Ser396/Ser404 (pS396/S404) partially restored abnormal Ca^2+^ activity and decreased pathological tau in 10‐ to 12‐month‐old JNPL3 mice.^4^ The improvement in Ca^2+^ activity correlated with decreased soluble pathological tau.^4^ Another tau mAb, 8B2, also targets pS396/S404, but has a different binding profile than 4E6.^17^ In the next step, we examined whether 8B2 also reduced pathological tau levels and rescued abnormal Ca^2+^ activity in motor cortical neurons in awake and behaving JNPL3 mice. JNPL3 mice were imaged on Day 0, followed by intravenous injections of two doses of either 8B2 (100 µg per dose) or IgG1 control 3 days apart (Days 1 and 4), followed by imaging again on Day 7 and brain extraction on Day 8 (Figure 7A). Ca^2+^ activity profiles were assessed before and after the acute antibody treatment, and brain tau levels were assessed at the end of the study. A subset of mice was injected intravenously with 8B2 or control IgG1 labeled with VivoTag 680XL to assess 8B2 target engagement. First, we examined the target engagement of 8B2 by immunostaining brain sections collected after imaging sessions. Substantially more 8B2 was detected in L2/3 motor cortical neurons compared to IgG1 control (Figure 7B). In addition, immunostaining results revealed that colocalization of PHF1 immunoreactivity with injected labeled‐8B2 signal was significantly more prevalent than such overlap of PHF1 staining and labeled‐IgG1 (Figure 7C, complete blots in Figure S4). Similarly, colocalization of CP27 immunoreactivity and injected labeled‐8B2 signal was also significantly more common than such overlap of CP27 staining and labeled‐IgG1 (Figure 7D). Furthermore, the acute intravenous 8B2 treatment significantly decreased soluble pS396/S404 (PHF1) and pT231, but did not affect total tau levels (CP27) in the LSS of brain homogenates (Figure 7E,F). We further applied sarkosyl extraction to enrich insoluble tau aggregates from LSS. Treatment with 8B2 significantly increased sarkysol‐insoluble total tau detected by CP27 (Figure 7E,F). Overall, acute 8B2 treatment decreased soluble p‐tau and increased insoluble total tau in motor cortex of JNPL3 mice.

Tauopathy mice exhibit gliosis, and the mouse IgG1 antibody subclass has been reported to have a low activating‐to‐inhibitory ratio (0.1),^25^ and is therefore unlikely to promote inflammation. Therefore, we evaluated the glia status of JNPL3 mice after either 8B2 or control IgG1 treatment. The antibody 8B2 showed a strong colocalization with microglia marker Iba1, whereas the control IgG1 barely overlapped with Iba1 (Figure S5A,B). Moreover, 8B2 treatment significantly decreased the Iba1‐positive area compared to IgG1 control–treated JNPL3 mice (Figure S5C). Within the microglia, 8B2 was bound to p‐tau (Figure S5D). In astrocytes, there was no difference in the colocalization of 8B2/GFAP vs IgG1/GFAP (Figure S5E,F). However, mice treated with 8B2 exhibited fewer GFAP‐positive astrocytes compared to IgG1‐treated mice (Figure S5G). In addition, the level of GFAP and Iba1 both decreased significantly in the forebrain lysates from mice treated with 8B2, compared to those treated with control IgG1 (Figure S5H–J, complete blots in Figure S6). Overall, the acute intravenous 8B2 treatment decreased microgliosis and astrogliosis in JNPL3 mice, which likely relates to its clearance of soluble pathological tau and the inhibitory characteristics of the IgG1 subclass on the immune response.^25^ Although 8B2 was detected bound to tau within microglia, that is unlikely to have a major effect on tau clearance because most of pathological tau is found within neurons, where antibodies can bind to it and promote its clearance, as shown here for 8B2 (Figure 7).^26^

### 3.6 Partially restored somatic Ca^2+^ activity in 6‐month‐old JNPL3 mice acutely treated with 8B2

**FIGURE 8:**
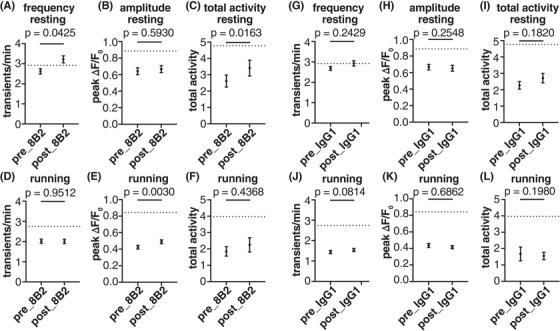
Abnormal Ca^2+^ activity is partially restored in L2/3 motor cortical neurons of JNPL3 mice after acute tau antibody 8B2 treatment. (A–F) Ca^2+^ activity frequency, peak amplitude, and total neuronal activity were analyzed in JNPL3 mice treated with 8B2 when animals were either resting or running on the treadmill. The Ca^2+^ activity in the same neuron before and after treatment was compared. In the resting condition, the frequency of Ca^2+^ transients increased significantly to WT level (dotted line). Total activity of Ca^2+^ transients also improved significantly after 8B2 treatment. In the running condition, peak amplitude of Ca^2+^ transients increased significantly after 8B2 treatment. A total of 233 neurons from 6 JNPL3 mice were analyzed. (G–F) Ca^2+^ activity in JNPL3 mice treated with control IgG1. The same analyses were conducted as in (A–F). Control IgG1 did not affect Ca^2+^ activity abnormalities. A total of 349 neurons from 7 JNPL3 mice were analyzed. Wilcoxon matched‐pair signed‐rank test in (A–F). WT, wild‐type.

Considering the excellent target engagement of the 8B2 antibody and its efficacy in reducing pathological tau, we tracked the Ca^2+^ activity in the same neuronal soma before and after 8B2 treatment, which was not performed in our previous study.^4^ In resting JNPL3 mice, Ca^2+^ transient frequency and total Ca^2+^ activity increased significantly after 8B2 treatment (Figure 8A,C). The fraction of hypoactive neurons decreased (38% vs 30%, before vs after; Figure S7A) and the fraction of hyperactive neurons increased (6% vs 12%, before vs after; Figure S7A). The peak amplitude of Ca^2+^ transients remained the same before and after 8B2 treatment (Figure 8B and S7B). In running JNPL3 mice, the frequency of Ca^2+^ transients and total Ca^2+^ activity were similar before and after 8B2 treatment (Figures 8D, S7A, and 8F), but peak amplitude of Ca^2+^ transients increased significantly after 8B2 treatment (Figure 8E and S7B).

Furthermore, we examined whether 8B2 treatment restored altered neuronal network activity during running in JNPL3 mice. Although there were trends of an increase in activated neurons and a decrease in suppressed neurons after acute 8B2 treatment, the changes did not reach statistical significance (Figure S7C–E). In addition, 8B2 treatment did not affect altered Ca^2+^ activity synchrony in either activated or suppressed neuronal populations, respectively (Figure S7F).

To test if the functional improvement was specific to 8B2 treatment, we analyzed Ca^2+^ activity profiles in a cohort of JNPL3 mice treated by control IgG1. Two doses of control IgG1 treatment did not alter the frequency or amplitude of Ca^2+^ transients, or total Ca^2+^activity in either resting or running JNPL3 mice (Figure 8G–L). Unlike 8B2 treatment, control IgG1 treatment increased the fractions of hypoactive and hyperactive neurons and decreased the fraction of normal neurons under resting condition (Figure S8A). During running, the fractions of these three different types of neurons remained the same before and after control IgG1 treatment (Figure S8A). Moreover, control IgG1 did not alter the pattern of neuronal responses to running (Figure S8B–D). In addition, we observed no change in Ca^2+^ activity synchrony among activated neurons or suppressed neurons during running after control IgG1 treatment (Figure S8E).

We also treated a cohort of WT mice with two doses of 8B2 to investigate if 8B2 treatment affected Ca^2+^ activity in healthy mice. No significant changes in frequency or amplitude of Ca^2+^ transients or total Ca^2+^ activity were observed in resting or running WT mice after 8B2 treatment (Figure S9A). In addition, the fractions of hypoactive, hyperactive, and normal neurons remained similar before and after 8B2 treatment (Figure S9B).

Taken together, these data show that two doses of 8B2 treatment partially restored somatic Ca^2+^ activity profiles in motor cortical neurons in JNPL3 tauopathy mice under resting and running conditions. However, this treatment did not restore the abnormal neuronal network activity in these mice. Nevertheless, this acute treatment paradigm and the assessment of neuronal functional readouts can be further optimized and utilized as a foundation to evaluate the therapeutic potential of tau antibodies in future studies.

## 4 DISCUSSION

In this study, we detected abnormal Ca^2+^ activity in motor cortical neurons of 6‐month‐old awake and behaving JNPL3 mice in their early stage of tauopathy. More pronounced changes in Ca^2+^ activity were observed in 6‐ compared to 12‐month‐old cohorts when comparing JNPL3 mice and their age‐matched wild‐type (WT) controls. In resting 6‐month‐old animals, a notable decrease in both peak amplitude and total activity of Ca^2+^ transients was observed in JNPL3 mice, compared to WT mice. Moreover, running JNPL3 mice exhibited a more pronounced reduction of Ca^2+^ transient frequency, amplitude, and total Ca^2+^ activity, compared to WT mice. In 12‐month‐old animals, only the peak amplitude of Ca^2+^ transients decreased significantly in resting or running JNPL3 mice, compared to WT mice. The attenuation of Ca^2+^ activity in young JNPL3 mice did not further deteriorate at 12 months of age, and total Ca^2+^ activity was decreased in older WT mice compared to their younger cohort. This lack of progression of neuronal deficits in the motor cortex from 6 to 12 months of age may be explained by their comparable cortical tau burden as measured by western blots.

We further analyzed the Ca^2+^ activity profiles of the 6‐month‐old cohorts in detail. A large fraction of neurons were hypoactive in JNPL3 mice. Treadmill running revealed that JNPL3 mice had less activated and more suppressed neurons compared to WT mice. Despite fewer activated neurons in JNPL3 mice, higher synchrony of Ca^2+^ activity was observed in resting animals, and disrupted engagement of neuronal Ca^2+^ activity during running. Altered excitatory synaptic transmission and enhanced intrinsic neuronal excitability were also observed, which likely contributed to the decreased Ca^2+^ activity and disrupted neuronal synchrony in JNPL3 mice. Finally, we attempted to improve neuronal and network function by acute tau antibody treatment. Two doses of tau antibody 8B2 partially restored Ca^2+^ activity profiles but not network activity in JNPL3 mice. The 8B2 antibody was taken up by neurons and microglia and co‐localized with total tau and phosphorylated tau. The treatment decreased soluble p‐tau, increased total insoluble tau, and attenuated microgliosis and astrogliosis in JNPL3 mice.

A major observation in this study was decreased Ca^2+^ activity in the motor cortical neurons of 6‐month‐old JNPL3 mice. This deficit was further exacerbated during running. Specifically, a larger fraction of neurons became silent or hypofunctional, and the remaining active neurons exhibited less Ca^2+^ activity. This indicates that the proper neuronal response to running behavior was disrupted in these tauopathy mice. The changes in Ca^2+^ transient amplitude and total Ca^2+^ activity were similar to our prior analyses of older 10‐ to 12‐month‐old JNPL3 mice, whereas changes in the frequency of Ca^2+^ transients differed from that of the prior report.^4^ This suggests persistent but evolving changes in neuronal activity with aging in tauopathy mice. Reduced neuronal firing rates or Ca^2+^ transient frequency has been reported in other mouse models at an early stage of tauopathy.^5^, ^6^ However, those studies did not explore changes in Ca^2+^ activity amplitude or total Ca^2+^ activity, especially in awake and running animals.

In addition to analyzing Ca^2+^ activity profiles, we also examined neuronal network activity in the younger cohorts. We are not aware of such prior analyses in tauopathy mice. Although neurons in tauopathy mice showed reduced activity, the synchrony of basal neuronal Ca^2+^ activity increased significantly in resting 6‐month‐old JNPL3 mice, which was not examined in our previous study in older animals.^4^ Increased neuronal synchrony was reported in hyperactive neurons in an Alzheimer's disease mouse model with amyloid‐beta (Aβ) deposits.^27^ However, neurons in Aβ mouse models usually exhibit hyperactivity,^6^, ^20^ which is in contrast to the hypoactivity in tauopathy models reported by us and others.^4^, ^5^, ^6^, ^28^, ^29^ Nevertheless, excessive neuronal synchrony can lead to elliptical‐like discharge in patients with Alzheimer's disease and mouse models.^13^ Therefore, we hypothesize that pathological tau may synergize with Aβ to increase synchronization of basal neuronal activity. When mice were running, neuronal Ca^2+^ activity synchrony also increased in JNPL3 mice. However, neurons with different responses to running showed diverging changes. Activated neurons were more synchronized, whereas suppressed neurons were less synchronized. These data indicate that JNPL3 mice had disrupted neuronal circuitry engagement in L2/3 motor cortex during running.

To further investigate the underlying mechanism causing reduced Ca^2+^ activity and altered network activity, we analyzed by electrophysiology cortical brain slices from tauopathy and control mice. Previously, accumulation of hyperphosphorylated tau was associated with reduced excitability of hippocampal neurons in young 3xTg mice.^30^ However, we did not observe a decrease in intrinsic excitability in L2/3 motor cortical neurons in 6‐month‐old JNPL3 mice. Instead, both fast‐spiking (FS)‐ and non‐FS neurons exhibited enhanced excitability. This difference is possibly due to different brain regions and animal models used in our versus that study. The excitability of FS neurons was slightly enhanced in JNPL3 mice. However, mutant tau did not change the input resistance, capacitance, or rheobase current of FS neurons. Therefore, the enhanced firing ability of FS neurons was likely associated with increased excitatory inputs onto those cells, specifically increased sponteneous excitatory post‐synaptic current (sEPSC) frequency. As detailed in the Results, layer 2/3 FS interneurons receive excitatory inputs from other cortical layers and thalamocortical neurons,^23^, ^25^ which may contribute to the increased frequency of sEPSCs in these FS interneurons. Consequently, increased FS neuronal firing may contribute to enhanced synchrony of neuronal activity, given the roles of FS interneurons in network oscillations.^31^ In contrast, non‐FS neurons in JNPL3 mice had significantly decreased excitatory synaptic transmission indicated by a decrease in sEPSC frequency, while exhibiting a greater increase in excitability. The mutant neurons displayed larger input resistance, likely due to a decrease in basal repolarization conductance. As a result, these neurons had depolarized resting membrane potential and fired action potentials (APs) at lower rheobase currents. Enhanced excitability in non‐FS neurons may be a compensatory response to decreased excitatory synaptic transmission onto them through homeostatic mechanisms.^24^ Nevertheless, these disproportional changes of excitability in FS (presumably inhibitory) and non‐FS neurons (presumably excitatory) may contribute to an imbalance between excitatory and inhibitory activity (E/I imbalance) and compromise local network activity in JNPL3 mice. Further studies on how pathological tau alters firing properties in different neuronal subtypes are needed. Because E/I balance modulates neuronal firing synchrony in physiological conditions,^12^, ^13^, ^32^ further exploration of how altered E/I balance disrupts neuronal population activity in pathological conditions like tauopathy is also needed to clarify circuit mechanisms of network dysfunction in behaving animals.

Finally, we evaluated the therapeutic efficacy of a tau antibody 8B2 in the 6‐month‐old cohort of JNPL3 mice. We used the same acute treatment paradigm as in our previous report on tau antibody 4E6 in 10‐ to 12‐month‐old JNPL3 mice.^4^ The 8B2 antibody targets the pS404 epitope of tau‐like 4E6, but their affinities and exact binding site differ substantially.^17^ Different from our analyses in Wu et al., we aligned images obtained before and after antibody treatment, and tracked the Ca^2+^ activity of individual neurons across different days, which enhances the power of the analyses. Two acute doses of 8B2 restored Ca^2+^ transient frequency and total Ca^2+^ activity in resting animals, and Ca^2+^ transient amplitude in running animals. However, it failed to rescue altered population patterns of Ca^2+^ activity and the network engagements to running in JNPL3 mice. These functional benefits were associated with decreased soluble p‐tau and increased insoluble tau. We have shown previously that 8B2 has a high affinity for paired helical filament (PHF)–enriched tau.^17^ Under this acute paradigm, the therapeutic benefit of 8B2 may in part relate to its ability to sequester insoluble tau, while promoting clearance of soluble tau. We have reported before for the 4E6 tau antibody that acute in vivo treatment primarily reduces soluble tau, whereas insoluble tau is not affected.^4^, ^33^, ^34^ The key difference between 8B2 and 4E6 is that the latter antibody has a low affinity for insoluble tau. In addition, 8B2 antibody was efficiently taken up by neurons and microglia and co‐localized with tau, indicating target engagement. Furthermore, treatment with the 8B2 antibody significantly decreased microgliosis and astrogliosis in JNPL3 mice. We have reported previously comparable tau clearing efficacy of mouse IgG1 versus IgG2a subclasses of a different tau antibody, both in mixed culture with all cell types and in vivo.^35^ IgG2a has the strongest effector function of all mouse antibody subclasses (activating‐to‐inhibitory ratio of 69).^25^ Hence, because the efficacy of these subclasses is the same, despite a very different activating‐to‐inhibitory ratio (0.1 vs 69), microglial phagocytosis of tau–antibody complex is unlikely to be a major factor in tau clearance. The reduced gliosis likely relates to improved neuronal health and function because of neuronal clearance of soluble pathological tau, and maybe in part due to the inhibitory effect of the IgG1 subclass on the immune response. As we have emphasized over the years, most of pathological tau is found within neurons and that is where the majority of antibody‐mediated tau clearance likely takes place.^26^ Altogether, these data support functional benefits of acute antibody 8B2 treatment in 6‐month‐old JNPL3 mice, supporting chronic studies on this antibody that should provide greater efficacy.

## 5 SUMMARY

In the JNPL3 tauopathy mouse model, reduction of neuronal Ca^2+^ activity was observed in the motor cortex of awake and behaving animals at the early stage of tauopathy that was exacerbated under the running condition, compared to age‐matched wild‐type controls. This neuronal deficit did not further deteriorate from 6 to 12 months of age, presumably because cortical tau burden did not increase between these ages. Furthermore, the tau pathology affected local neuronal circuitry, resulting in increased synchrony of basal Ca^2+^ activity and altered running‐related neuronal responses in L2/3 of the primary motor cortex. Underlying mechanisms may link to impairments of excitatory synaptic transmission and altered E/I balance in this region resulting from accumulation of pathological tau. Tau antibody treatment cleared soluble phosphorylated tau, increased insoluble total tau, and ameliorated gliosis while partially restoring abnormal Ca^2+^ activity profile, highlighting the importance of functional assessment when evaluating the therapeutic efficacy of anti‐tau therapies.

## AUTHOR CONTRIBUTIONS

Changyi Ji and Einar M. Sigurdsson conceived the project and wrote the article. Changyi Ji and Xiaofeng Yang performed most of the experiments and related analyses. Mohamed Eleish analyzed some of the electrophysiological data. Yixiang Jiang prepared the labeled 8B2 antibody and control IgG. Amber M. Tetlow performed immunofluorescent staining of the mouse brains. Soomin C. Song provided guidance on electrophysiological recording and data analyses. Alejandro Martín‐Ávila, Qian Wu, Yanmei Zhou, and Wenbiao Gan provided guidance on two‐photon calcium imaging experiments. Yan Lin maintained the animal colonies. All authors had the opportunity to edit the article. Einar M. Sigurdsson supervised the project.

## CONFLICT OF INTEREST STATEMENT

E.M.S. is an inventor on a patent application that describes the initial characterization of the 8B2 antibody and is assigned to New York University. The authors declare that they have no other competing interests. Author disclosures are available in the Supporting Information.

## ETHICS STATEMENT

All procedures were approved by the Institutional Animal Care and Use Committee (IACUC) of the New York University Grossman School of Medicine, and are in accordance with National Institutes of Health (NIH) Guidelines, which meet or exceed the Animal Research: Reporting of In Vivo Experiments (ARRIVE) guidelines. We thank Dr. Peter Davies (Albert Einstein College of Medicine and Long Island Jewish Medical Center, Litwin‐Zucker Research Center at Feinstein Institutes for Medical Research) for the tau antibodies PHF1 and CP27, which were obtained subsequently from Drs. Philippe Marambaud and Jeremy Koppel at the Feinstein Institutes.

## Supporting information

Supporting Information

Supporting Information

## Data Availability

All data needed to evaluate the conclusions in the article are present herein and/or the Supplemental Materials.
