# Supplementary material for: Neuronal hypofunction and network dysfunction in a mouse model at an early stage of tauopathy
Source: Alzheimers Dement. 2024 Oct 5;20(11):7954–70. doi: 10.1002/alz.14273 (PMC11567809; doi:10.1002/alz.14273)
Supplement: Supplementary file 1 — Supporting Information [file ALZ-20-7954-s001.pdf]

Figure Name		Statistical Methods
Figure 1	C, D	Two-way ANOVA with Fisher's LSD multiple comparisons test
Figure 2	A	Kolmogorov-Smirnov test
	C	Two-way ANOVA with Tukey's multiple comparisons test
	D	Mann-Whitney U test
Figure 3	A, D	Pearson's correlations
	B, C, E, F	Unpaired t test
Figure 4	E	Multiple unpaired t test
Figure 5	B, D	Unpaired t test
Figure 6	B, F	Two-way ANOVA with Sidak's multiple comparisons test
	D, H, I, J	Unpaired t test
Figure 7	B, C, D, F	Unpaired t test
Figure 8	A - L	Wilcoxon matched-pair signed rank test
Supplemental figure 1	B, D, H	Unpaired t test
Supplemental figure 3	A, C, E, G	Mann-Whitney U test
	B, D, F, H	Kolmogorov-Smirnov test
Supplemental figure 5	B, C, F, G, H, I, J	Unpaired t test
Supplemental figure 7	B	Kolmogorov-Smirnov test
	D	Multiple unpaired t test
	F	Unpaired t test
Supplemental figure 8	C	Multiple unpaired t test
	E	Unpaired t test
Supplemental figure 9	A	Wilcoxon matched-pair signed rank test

Supplemental Table 1. Statistical methods used in figures

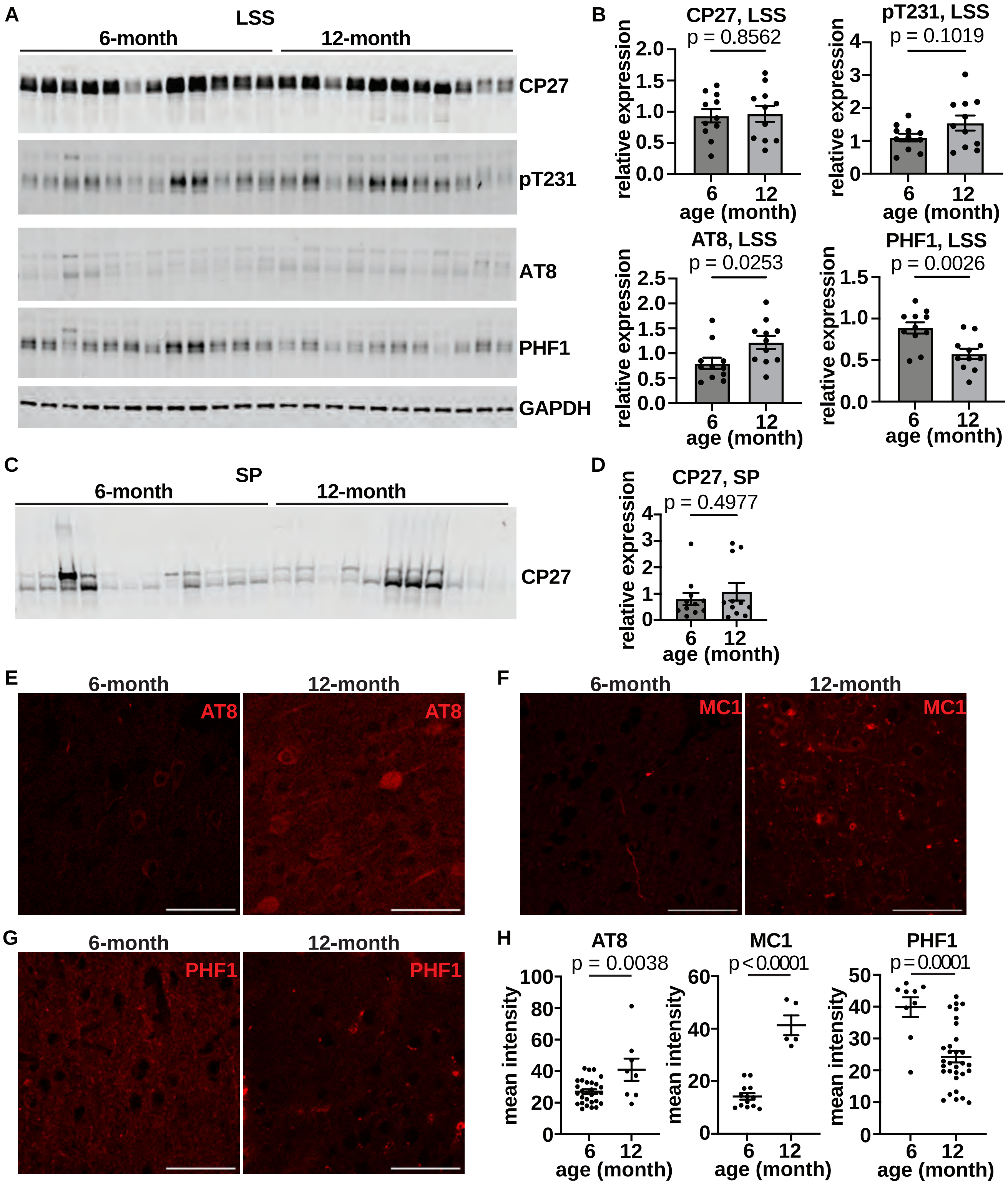

Supplemental Figure 1. Progressive tau pathology in JNPL3 mice. (A) Western blots of total tau and phosphorylated tau in low-speed supernatant (LSS) of brain homogenates from forebrain cortexes of 6-month-old and 12-month-old JNPL3 mice. Total human tau was recognized by CP27 antibody, phosphorylated tau (p-tau) was detected by pT231, AT8 (pS202/T205) or PHF1 (pS396/S404), and GAPDH was used as loading control. (B) Quantification of tau levels in (A). Total tau and p-tau were normalized to GAPDH, and then compared between 6-month-old and 12-month-old JNPL3 mice. (C) Western blots of total tau in sarkosyl insoluble (SP) fractions. (D) Quantification of tau levels in (C). (E-G) Representative images of p-tau (recognized by AT8 and PHF1) and misfolded tau (recognized by MC1) immunostaining in the primary motor cortex of JNPL3 mice. Scale bar: 50 µm. (H) Quantification of the mean fluorescence intensity of p-tau or misfolded tau. Unpaired t test in B, D and H.

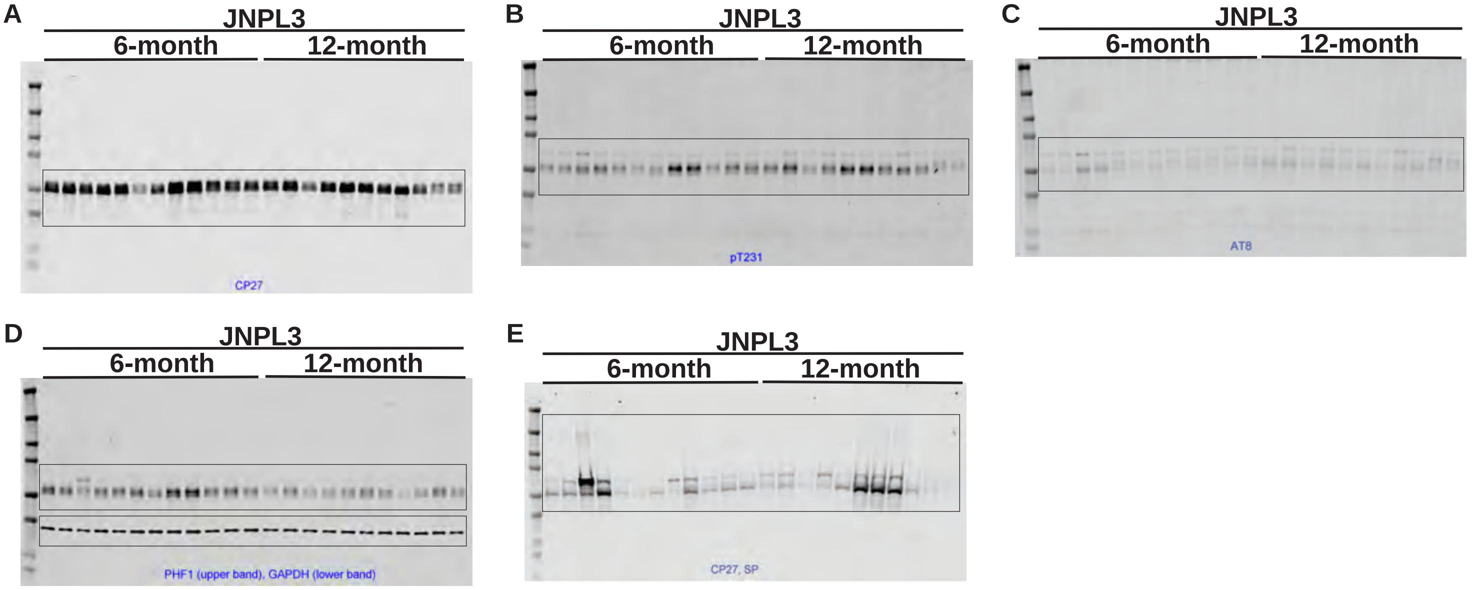

Supplemental Figure 2. Complete western blots (A–E) from Figure S1A. Boxes denote the blot sections shown in Figure S1A.

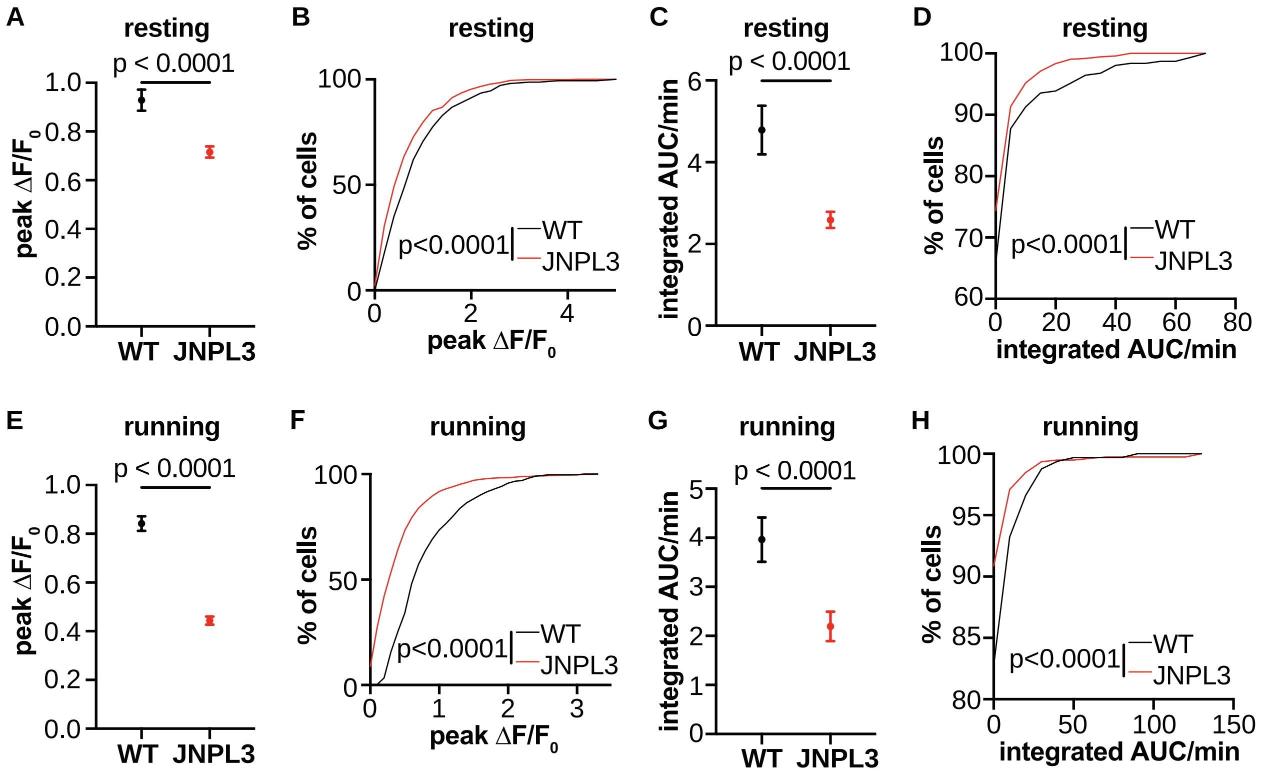

Supplemental Figure 3. Decreased Ca^{2+} activity in L2/3 motor cortical neurons of 6-month-old JNPL3 mice. (A) The maximum Ca^{2+} transient amplitude in active neurons (transients/min > 0) decreased in resting JNPL3 mice, compared to WT mice. (B) Cumulative frequency distribution of (A). (C) The total activity (integrated Ca^{2+} activity per min) in active neurons in resting JNPL3 mice, which decreased compared to WT mice. (D) Cumulative frequency distribution of (C). (E) The maximum Ca^{2+} transient amplitude in active neurons decreased in running JNPL3 mice, compared to WT mice. (F) Cumulative frequency distribution of (E). (G) The total activity (integrated Ca^{2+} activity per min) in active neurons in running JNPL3 mice, which decreased compared to WT mice. (H) Cumulative frequency distribution of (G). In resting condition, a total of 301 neurons from WT animals and 733 neurons from JNPL3 mice were analyzed. Under running condition, a total of 325 neurons from WT animals and 802 neurons from JNPL3 mice were analyzed. Mann-Whitney U test in A, C, E and G. Kolmogorov-Smirnov test in B, D, F and H.

	**Ca^{2+} activity profiles**	**6 months**		**12 months**	
		**JNPL3 vs WT**		**JNPL3 vs WT**	
		**resting**	**running**	**resting**	**running**
Animal level	Ca^{2+} transient frequency	↔	↓	↔	↔
	Ca^{2+} transient amplitude	↓	↓	↓	↓
	integrated Ca^{2+} transient AUC	↓	↓	↔	↔
	Ca^{2+} activity correlations	↑	↔	NA	NA
Neuronal level	fraction of hypoactive neurons	↔	↑	NA	NA
	Ca^{2+} transient frequency of active neurons	↔	↓	NA	NA
	Ca^{2+} transient amplitude of active neurons	↓	↓	NA	NA
	integrated Ca^{2+} transient AUC of active neurons	↓	↓	NA	NA
	Ca^{2+} activity correlations	↑	↑	NA	NA
	fraction of activated neurons	NA	↓	NA	NA
	fraction of suppressed neurons	NA	↑	NA	NA
	Ca^{2+} activity correlations of activated neurons	NA	↑	NA	NA
	Ca^{2+} activity correlations of suppressed neurons	NA	↓	NA	NA

Supplemental Table 2. Summary of results by in vivo two photon Ca^{2+} imaging. Ca^{2+} transient frequency, spike number per second; Ca^{2+} transient amplitude, peak $\Delta F/F_0$; integrated Ca^{2+} transient AUC, Ca^{2+} transient AUC per minute; Ca^{2+} activity correlations, Pearson's correlation of $\Delta F/F_0$ over time between neuronal pairs in a given field of view.

Cell type	Genotype	Resting membrane potential (mV)	Rm (MΩ)	Cm (pF)	Rheobase current (pA)
FS	WT (n = 5)	-73.05 ± 0.71	153.80 ± 26.98	28.8 ± 2.87	159.80 ± 51.75
	JNPL3 (n = 10)	-70.4 ± 1.15	191.30 ± 33.22	26.9 ± 2.63	160.90 ± 32.57
	p-value (unpaired t test)	0.1496	0.4774	0.6632	0.9861
non-FS	WT (n = 14)	-75.07 ± 1.37	78.36 ± 17.69	86.4 ± 8.54	283.85 ± 24.74
	JNPL3 (n = 6)	-69.07 ± 2.19	151.00 ± 14.20	46.8 ± 5.95	113.33 ± 18.91
	p-value (unpaired t test)	0.0291	0.0216	0.0101	<0.0001

Supplemental Table 3. Electrophysiological parameters of motor cortical L2/3 neurons in WT and JNPL3 mice. FS, fast-spiking neurons; non-FS, non fast-spiking neurons; Rm, input resistance; Cm, cell capacitance; Rheobase current, the minimal inject current to elicit an action potential.

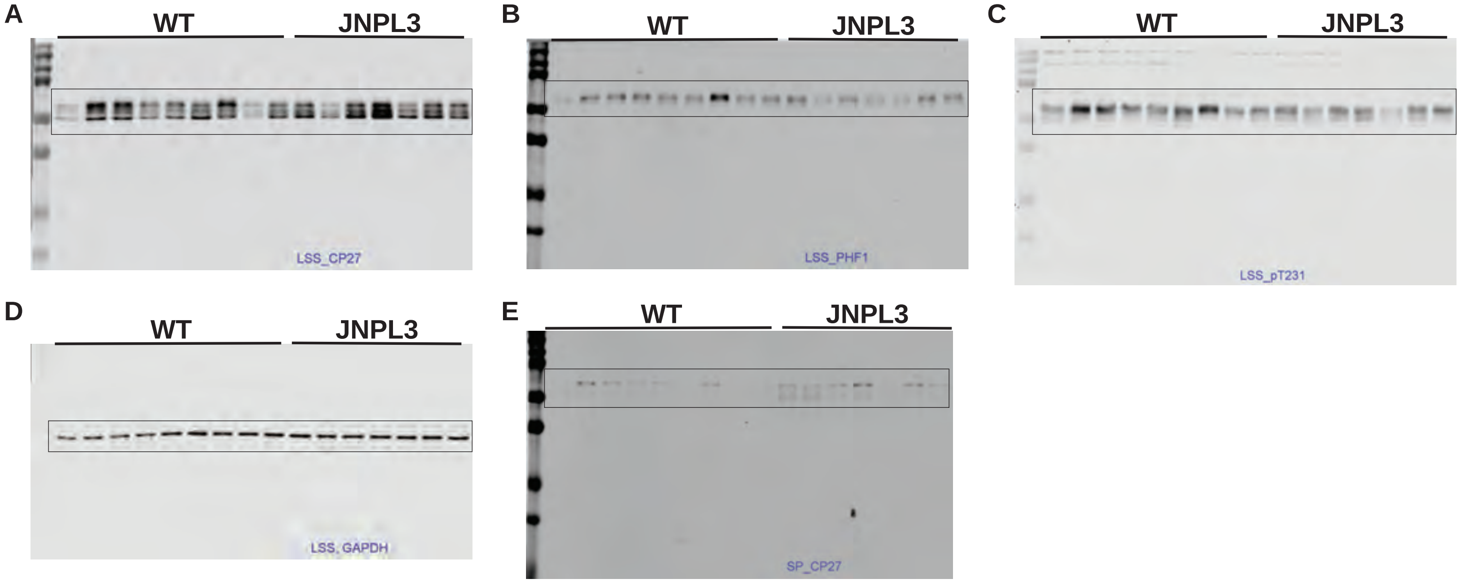

Supplemental Figure 4. Complete western blots (A–E) from Figure 7C. Boxes denote the blot sections shown in Figure 7C.

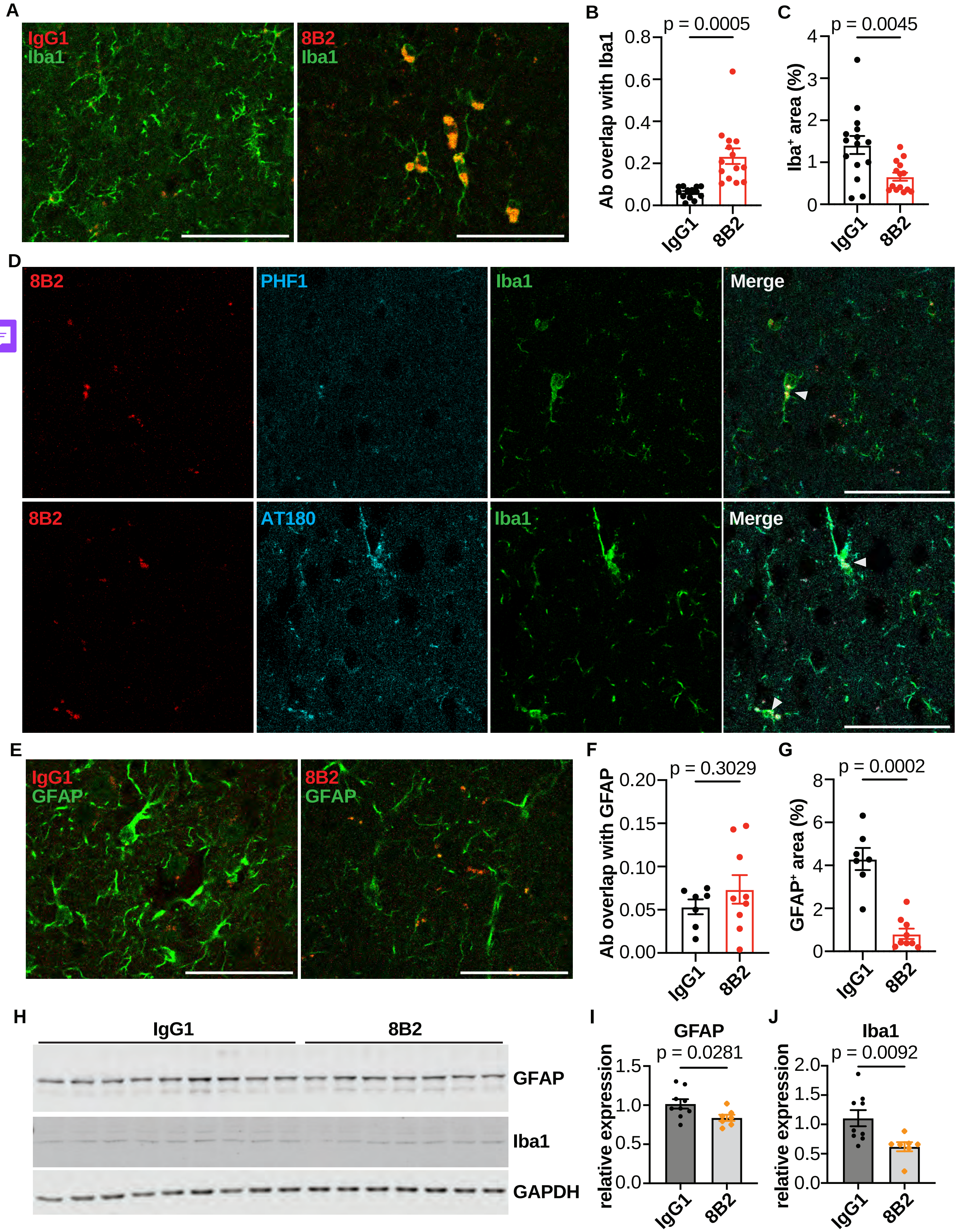
A
IgG1
Iba1
8B2
Iba1
B
p = 0.0005
Ab overlap with Iba1
0.8
0.6
0.4
0.2
0.0
IgG1
8B2
C
p = 0.0045
Iba+ area (%)
4
3
2
1
0
IgG1
8B2
D
8B2
PHF1
Iba1
Merge
8B2
AT180
Iba1
Merge
E
IgG1
GFAP
8B2
GFAP
F
p = 0.3029
Ab overlap with GFAP
0.20
0.15
0.10
0.05
0.00
IgG1
8B2
G
p = 0.0002
GFAP+ area (%)
8
6
4
2
0
IgG1
8B2
H
IgG1
8B2
GFAP
Iba1
GAPDH
I
GFAP
p = 0.0281
relative expression
1.5
1.0
0.5
0.0
IgG1
8B2
J
Iba1
p = 0.0092
relative expression
2.0
1.5
1.0
0.5
0.0
IgG1
8B2

Supplemental Figure 5. Attenuation of gliosis and accumulation of tau antibody 8B2 in microglia in the motor cortex of 6-month-old JNPL3 mice. These analyses were conducted on the same brains that were analyzed in Figure 7. (A) Coronal brain sections were stained with microglia marker Iba1. Colocalization between Iba1 and 8B2 was observed. (B) Overlapping signals between injected 8B2 and Iba1 staining were significantly more prevalent than those between IgG1 and Iba1. (C) Mice treated intravenously with 8B2 had less Iba1 reactive area than mice treated with control IgG1. (D) Colocalization of injected 8B2 and phospho-tau staining (recognized by PHF1 or AT180) in Iba1 positive microglia. (E) 8B2 or IgG1 had very limited overlap with astrocyte marker GFAP. (F) GFAP and 8B2 colocalization did not differ from GFAP and IgG1 colocalization. (G) Mice treated with 8B2 had less GFAP reactive area than IgG1 control treated mice. A, D and E show representative images from a mouse treated with either 8B2 or IgG1. (H) Western blots of GFAP and Iba1 in all animals. (I) Quantification of GFAP expression normalized to GAPDH levels. Mice treated with 8B2 had less GFAP expression than IgG1 control treated mice. (J) Quantification of Iba1 expression normalized to GAPDH levels. Mice treated with 8B2 had less Iba1 expression than IgG1 control treated mice. A total of 9 JNPL3 mice received intravenous IgG1 injections and 7 JNPL3 mice were injected with 8B2. Unpaired t test in B, C, F, G, H, and I. Colocalization was assessed by Mander's coefficiency. Scale bar, 50 µm.

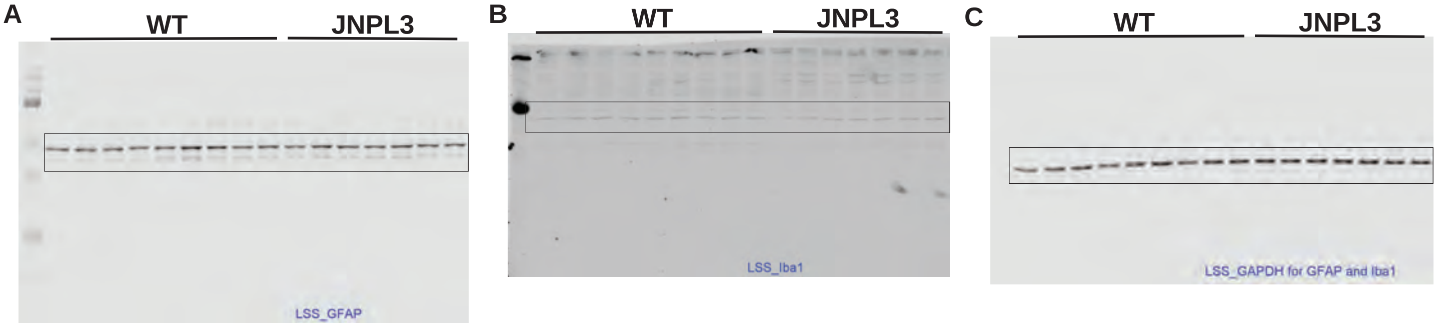

Supplemental Figure 6. Complete western blots (A–C) from Figure S5H. Boxes denote the blot sections shown in Figure S5H.

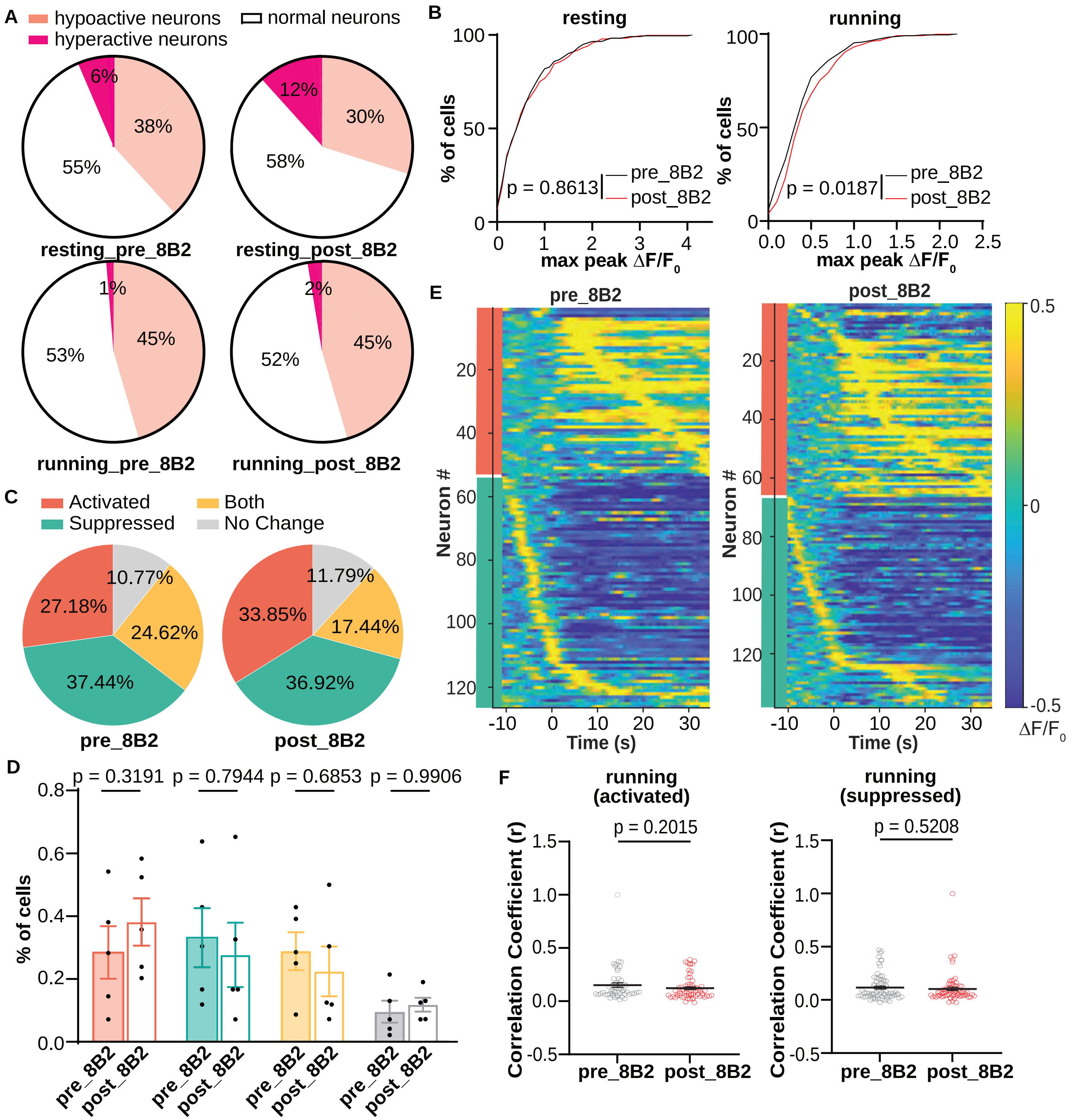

Supplemental Figure 7. 8B2 treatment failed to correct running-related neuronal Ca^{2+} activity abnormalities in L2/3 motor cortical neurons of JNPL3 mice. (A) The fractions of hypoactive neurons decreased in resting JNPL3 mice but remained unchanged in running mice after 8B2 treatment. (B) Cumulative frequency distribution of the maximal Ca^{2+} transient amplitude in neurons before and after 8B2 treatment. The distribution of maximal Ca^{2+} transient amplitude significantly changed during running after 8B2 treatment. (C) Fractions of neurons (195 neurons) categorized by their response to running before and after 8B2 treatment. (D) Fractions of neurons with each response to running in JNPL3 mice (5 animals) before and after 8B2 treatment. Each dot represents an animal. (E) Activity patterns of neurons which were either activated or suppressed during running before and after 8B2 treatment. (F) Correlation index of activated and suppressed neurons before and after the 8B2 treatment. Each dot represents the correlation index of a given neuron. Kolmogorov-Smirnov test in B. Unpaired t-tests in D and F.

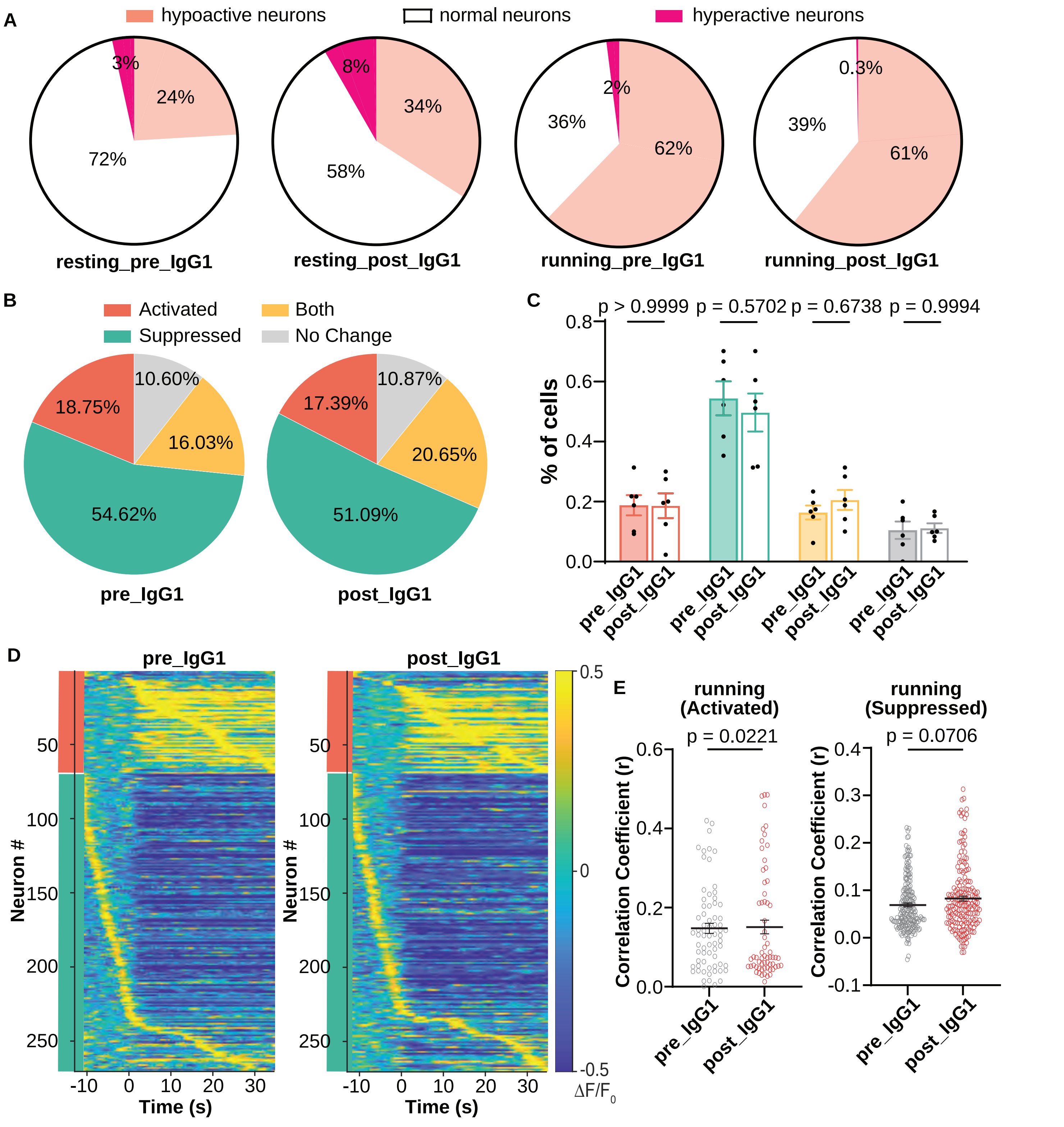

Supplemental Figure 8. No change or exacerbated abnormal Ca^{2+} activity in L2/3 motor cortical neurons of JNPL3 mice treated with IgG1 controls. (A) The fractions of hypoactive neurons increased in resting JNPL3 mice or no change in running JNPL3 mice after IgG1 treatment. (B) Ratios of neurons (368 neurons) categorized by their response to running before and after IgG1 treatment. (C) Fractions of neurons with each response to running in JNPL3 mice (6 animals) before and after IgG1 treatment. Each dot represents an animal. (D) Activity patterns of neurons with either activated or suppressed response to running before and after IgG1 treatment. (E) Correlation index of activated and suppressed neurons before and after IgG1 treatment. Each dot represents the correlation index of a given neuron. Unpaired t tests in C and E.

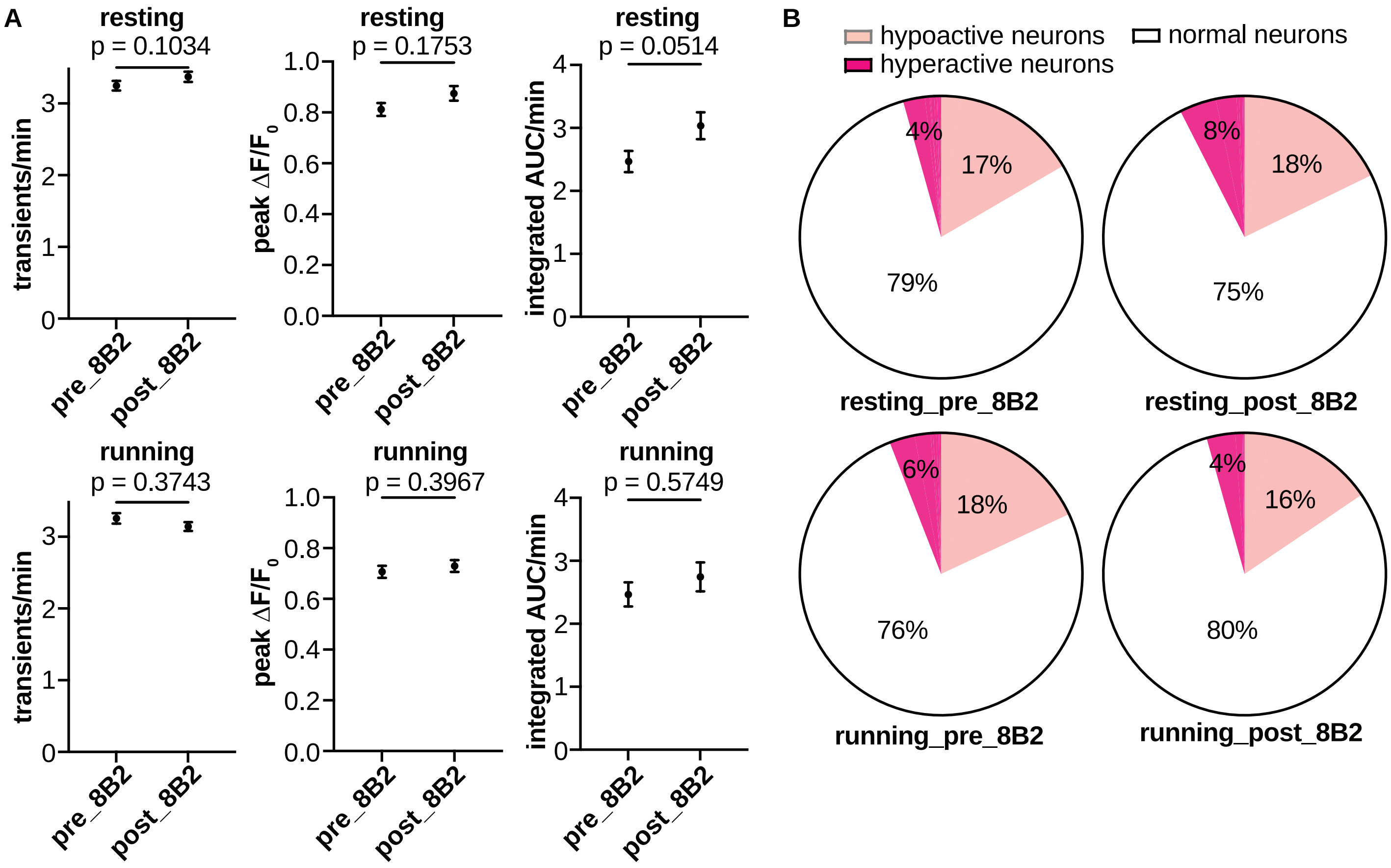

Supplemental Figure 9. Somatic Ca^{2+} activity in L2/3 motor cortex before and after 8B2 injection in 12-month-old WT mice. (A) Ca^{2+} activity frequency, amplitude, and total neuronal activity were analyzed in 12-month-old WT mice when animals were either resting or running on the treadmill. No significant change of Ca^{2+} activity was observed in WT neurons before vs after 8B2 treatment. (B) The fractions of hypoactive neurons remained unchanged in resting or running WT mice before and after 8B2 treatment. A total of 760 neurons from 5 WT mice were included in the analyses. Wilcoxon matched-pairs signed rank test in A.
